# Checkpoint kinase 2 controls insulin secretion and glucose homeostasis

**DOI:** 10.1038/s41589-023-01466-4

**Published:** 2023-11-09

**Authors:** Angie Chi Nok Chong, J. Jeya Vandana, Ginnie Jeng, Ge Li, Zihe Meng, Xiaohua Duan, Tuo Zhang, Yunping Qiu, Raimon Duran-Struuck, Kimberly Coker, Wei Wang, Yanjing Li, Zaw Min, Xi Zuo, Neranjan de Silva, Zhengming Chen, Ali Naji, Mingming Hao, Chengyang Liu, Shuibing Chen

**Affiliations:** 1 Department of Surgery, Weill Cornell Medicine, New York City, NY USA; 2 Center for Genomic Health, Weill Cornell Medicine, New York City, NY USA; 3 Tri-Institutional PhD Program in Chemical Biology, New York City, NY USA; 4 Department of Urology, Indiana University School of Medicine, Indianapolis, IN USA; 5 Department of Medicine, Weill Cornell Medicine, New York City, NY USA; 6 Department of Microbiology and Immunology, Weill Cornell Medicine, New York City, NY USA; 7 Department of Medicine, Fleischer Institute for Diabetes and Metabolism, Albert Einstein College of Medicine, Bronx, NY USA; 8 Department of Pathobiology, University of Pennsylvania School of Veterinary Medicine, Philadelphia, PA USA; 9 Department of Surgery, University of Pennsylvania School of Medicine, Philadelphia, PA USA; 10 Department of Population Health Sciences, Weill Cornell Medicine, New York City, NY USA; 11 Department of Biochemistry, Weill Cornell Medicine, New York City, NY USA; 12 Department of Biological Sciences, Bronx Community College, City University of New York, Bronx, NY USA

**Keywords:** Small molecules, Screening, Metabolism, Metabolomics, Cell signalling

## Abstract

After the discovery of insulin, a century ago, extensive work has been done to unravel the molecular network regulating insulin secretion. Here we performed a chemical screen and identified AZD7762, a compound that potentiates glucose-stimulated insulin secretion (GSIS) of a human β cell line, healthy and type 2 diabetic (T2D) human islets and primary cynomolgus macaque islets. In vivo studies in diabetic mouse models and cynomolgus macaques demonstrated that AZD7762 enhances GSIS and improves glucose tolerance. Furthermore, genetic manipulation confirmed that ablation of *CHEK2* in human β cells results in increased insulin secretion. Consistently, high-fat-diet-fed *Chk2*^*−*/*−*^ mice show elevated insulin secretion and improved glucose clearance. Finally, untargeted metabolic profiling demonstrated the key role of the CHEK2–PP2A–PLK1–G6PD–PPP pathway in insulin secretion. This study successfully identifies a previously unknown insulin secretion regulating pathway that is conserved across rodents, cynomolgus macaques and human β cells in both healthy and T2D conditions.

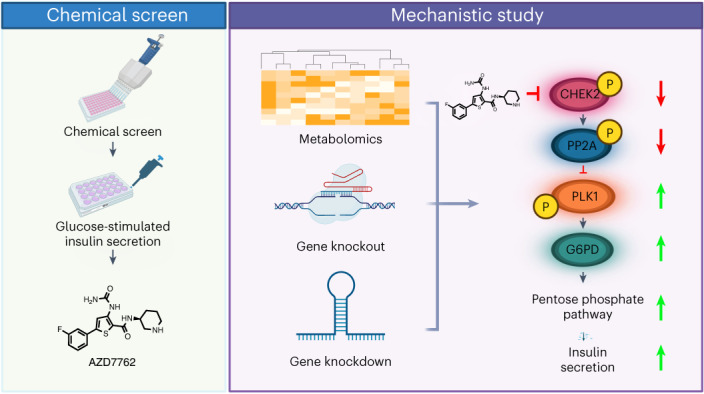

## Abstract (web summary)

A chemical screen identified a small molecule inhibitor of CHEK2 that boosts insulin secretion in human β cells, including those from both healthy and type 2 diabetic human islets, as well as in diabetic mouse models and cynomolgus macaques.

## Main

Insulin secretion by pancreatic β cells has a pivotal role in maintaining glucose homeostasis, and impaired insulin secretion is a hallmark of β cell failure in type 2 diabetes. The primary physiological secretagogue of β cells is glucose. Postprandially increased glucose molecules are taken up by β cells through GLUT1/GLUT2 glucose transporters, which allows glucose to enter the glycolytic pathway, tricarboxylic acid (TCA) cycle and the oxidative phosphorylation pathway in the mitochondria. The production of ATP through these processes increases ATP/ADP ratio, which leads to the closure of K_ATP_ channel. This is followed by membrane depolarization, the opening of the voltage-gated calcium channel and an increase in intracellular Ca^2+^ levels, ultimately triggering insulin granule exocytosis. This canonical glucose-stimulated insulin secretion (GSIS) triggering pathway involves the closure of K_ATP_ channels, which is primarily responsible for the rapid release of insulin within minutes upon glucose stimulation. The amplifying pathway allows sustained secretion of insulin and can be activated even when intracellular Ca^2+^ levels have reached the maximal levels. Together, the triggering and amplifying pathways of insulin secretion allow for initiation and fine-tuning of insulin secretory response to glucose and nutrients, as well as other factors, such as paracrine hormones—glucagon-like peptide-1 (GLP-1) and somatostatin (SST)^1–4^.

Several major metabolic cycles that have been shown to regulate GSIS include pyruvate–malate cycle, pyruvate–citrate cycle, pyruvate–isocitrate cycle^5^, phosphoenolpyruvate (PEP) cycle^6^, TCA cycle, mitochondrial oxidative phosphorylation and glycerolipid-free-fatty-acid (GL/FFA) cycle^1,2,4,7^. Potential crosstalk between metabolic cycles allows for a well-coordinated response of insulin secretion that ultimately converges to common downstream events that trigger exocytosis. Over the years, researchers have identified many metabolic mediator signals generated from these pathways that are critical for GSIS. These include ATP generated from the PEP cycle, TCA cycle and mitochondria oxidative phosphorylation; cytosolic NADPH from the pyruvate–malate cycle and pyruvate–isocitrate cycle; monoacylglycerols from GL/FFA, etc^1,2^. Evidence suggests that these intermediates influence the insulin secretion process at various steps and are responsible for tuning the overall strength of the cascade leading up to exocytosis. Because clinical studies suggested that both the triggering and amplifying phases are impaired in type 2 diabetes^8^, a detailed characterization and molecular understanding of insulin secretion cascades would help identify new target and strategy for therapy.

Chemical screens offer an unbiased approach to identify chemical tools to dissect biological processes related with human β cells. Most screens have focused on β cell identities and survival. Other screens focused on specific pathways and molecular targets that are known to regulate β-cell functions. Few studies^9^ have focused on insulin secretion, partially due to the lack of a high-throughput approach to monitor insulin secretion. In 2015, a proinsulin-NanoLuc fusion reporter was developed to allow real-time monitoring of insulin secretion^9^. Using this type of reporter, chemical screens were performed to identify compounds that improve insulin secretion^9,10^. However, the in vivo activity and molecular mechanism of these hit compounds are largely unknown.

Here we performed a focused chemical screen and identified AZD7762, which significantly increased insulin secretion from mouse and human β cells stimulated with high glucose. Using pharmacological and genetic approaches, we systematically confirmed the role of CHEK2 function in insulin secretion of EndoC-βH1 cells, healthy and T2D human islets, cynomolgus macaque islets, as well as in chow-fed, high-fat-diet (HFD)-fed and genetically obese leptin-deficient *Lep*^*ob*^^/^^*ob*^ (*ob*/*ob*) mice. By combining chemical screening, pharmacological, genetic and metabolomics approaches, we discovered a previously unknown role of the CHEK2–PP2A–PLK1–glucose-6-phosphate dehydrogenase (G6PD)–pentose phosphate pathway (PPP) in insulin secretion.

## Results

### Small molecule AZD7762 enhances insulin secretion

**Fig. 1:**
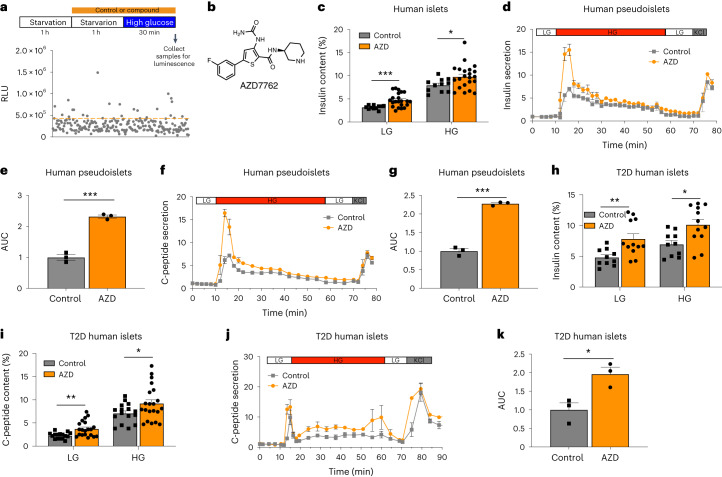
A focused chemical screen identified AZD7762 that increases glucose-stimulated insulin secretion of mouse and human islets. **a**, Schematic diagram of the chemical screen. **b**, Chemical structure of AZD7762. **c**, Static GSIS of intact human islets in the presence of control or 1 µM AZD7762. Low glucose (LG), 2 mM glucose (*P* = 0.0001); High glucose (HG), 20 mM glucose (*P* = 0.013). *n* = 11 (control) and *n* = 22 (AZD7762) biological replicates. **d**,**e**, Dynamic GSIS (**d**) and AUC (**e**) of human pseudoislets in the presence of control or 1 µM AZD776 (*P* = 0.0008). *n* = 3 biological replicates for each group. The data were normalized to baseline. **f**,**g**, Dynamic GSCS (**f**) and AUC (**g**, *P* = 0.0007) of human pseudoislets in the presence of control or 1 µM AZD7762. *n* = 3 biological replicates. The data were normalized to baseline. **h**, Static GSIS of T2D human islets in the presence of control or 1 µM AZD7762. LG, 2 mM glucose (*P* = 0.005); HG, 20 mM glucose (*P* = 0.008). *n* = 10 (control) and *n* = 12 (AZD7762) biological replicates. **i**, Static GSCS of T2D human islets in the presence of control or 1 µM AZD7762. LG, 2 mM glucose (*P* = 0.001); HG, 20 mM glucose (*P* = 0.034). *n* = 16 (control) and *n* = 21 (AZD7762) biological replicates. **j**,**k**, Dynamic GSCS (**j**) and AUC (**k**, *P* = 0.022) of T2D human islets in the presence of control or 1 µM AZD7762. *n* = 3 biological replicates. The data were normalized to baseline. Data represent the mean ± s.e.m. For **c**, **e**, **g**–**i** and **k**, *P* values of figures were calculated by two-sided Student’s *t*-test. Statistical significance: *P < 0.05, **P < 0.01, ***P < 0.001. Source data

**Extended Data Fig. 1:**
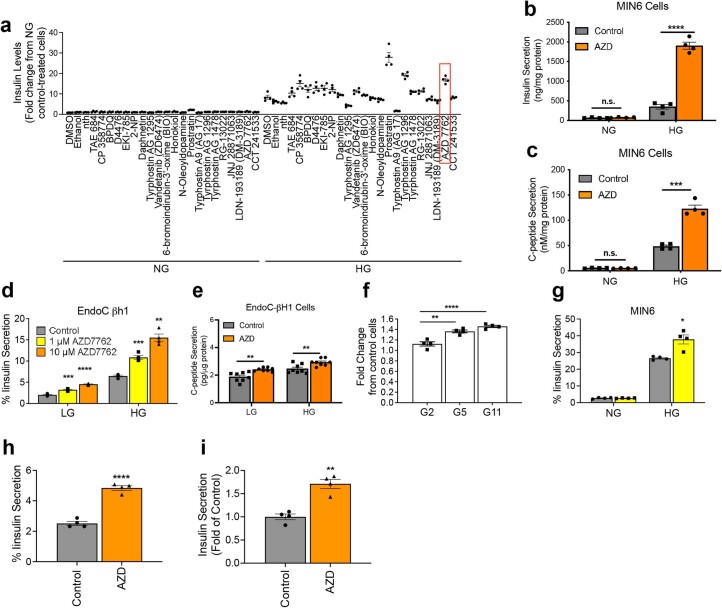
AZD7762 increases insulin secretion of β cells. (**a**) Primary screening data.(**b**) GSIS of MIN6 cells in the presence of control or 10 µM AZD7762. NG: 0 mM glucose; HG: 20 mM glucose. n = 4 biological replicates. *P*-value = 0.00005. (**c**) Glucose-stimulated C-peptide secretion (GSCS) of MIN6 cells in the presence of control or 10 µM AZD7762. NG: 0 mM glucose; HG: 20 mM glucose. *P*-value = 8.87E-04. n = 4 biological replicates. (**d**) GSIS of EndoC-βH1 cells in the presence of 0, 1 or 10 µM AZD7762. LG: 0.5 mM glucose; HG: 20 mM glucose. *P*-value: LG 1 µM AZD7762, p = 0.0001; LG 10 µM AZD7762, p = 6.75E-05; HG 1 µM AZD7762, p = 0.0008; HG 10 µM AZD7762, p = 0.00578. n = 4 biological replicates. (**e**) GSCS of human EndoC-βH1 cells in the presence of control or 10 µM AZD7762. LG: 0.5 mM glucose (*P*-value = 0.002); HG: 20 mM glucose (*P*-value = 0.0063). n = 4 biological replicates. (**f**) AZD7762 increases insulin secretion of NLuc-MIN6 cells in the presence of 2 (G2), 5 (G5) (*P*-value = 0.004), or 11 mM (G11) (*P*-value = 6.20E-050.001) glucose. n = 4 biological replicates. (**g**) GSIS of MIN6 cells in the presence of control or 1 µM AZD7762. NG: 0 mM glucose; HG: 20 mM glucose. *P*-value = 0.023. n = 4 biological replicates. (**h**) Insulin secretion during GSIS of EndoC-βH1 cells treated with control or 1 µM AZD7762 for 24 hours. *P*-value = 0.00003. n = 4 biological replicates. (**i**) Insulin secretion during a 2-hour period in the presence of control or 10 µM AZD7762. *P*-value = 0.002. n = 4 biological replicates. For b, c, e, g–i, *P*-value of figures were calculated by two-sided Student’s *t*-test. For d & f, *P*-value was calculated by one-way ANOVA (Dunnett′s test). Data represent the Mean ± SEM. Statistical significance *p* < 0.05, *; *p* < 0.01, **; *p* < 0.001, ***, *p* < 0.0001, ****; n.s. for not significantly different. Source data

To identify small molecules that acutely enhance insulin secretion from β cells, we performed a focused chemical screen using the mouse insulinoma β cell line (MIN6) carrying a proinsulin-NanoLuc reporter (NLuc-MIN6)^9^ with chemicals from an in-house chemical library containing 223 compounds targeting different signaling pathways (Supplementary Tables 1 and 2). After 1 h of treatment, cells were used for GSIS using luminescence signals as a surrogate for insulin levels (Fig. 1a). The compounds that enhanced luciferase signals under high glucose by more than 1.5-fold from the mean were chosen as primary hits. We further confirmed the activities of the hit compounds with a subsequent GSIS experiment and observed that 62% of the primary hits elevated high glucose-stimulated insulin secretion (Extended Data Fig. 1a). Among the 21 primary hits, 19 compounds were found to target signaling pathways that have been previously implicated to be involved in regulating insulin secretion, insulin sensitivity, β-cell proliferation, β-cell apoptosis or glucose metabolism (Supplementary Table 3). This finding validates the effectiveness of our screening platform. Among the 21 primary hits, AZD7762 (1), prostratin and tyrphostin AG1296 are the top three compounds that have the strongest effects on insulin secretion. Prostratin is known to activate protein kinase C, which has a well-documented role in regulating insulin secretion in β cells^11^. Tyrphostin AG1296 is an inhibitor of platelet-derived growth factor receptor (PDGFR). PDGFR belongs to receptor tyrosine kinases, whose activation has been shown to be involved in β-cell exocytosis^12,13^ and proliferation^14^. AZD7762 (Fig. 1b) is a competitive dual CHEK1/CHEK2 inhibitor^15^. The role of CHEK2 in insulin secretion is largely unknown. Because AZD7762 has been used in human clinical trials^16^, this small molecule represents a useful tool for further elucidating the role of CHEK2 in insulin secretion in vitro and in vivo. AZD7762 increased luminescent signals from NLuc-MIN6 cells in a dose-dependent manner in the presence of 20 mM d-glucose, but not in the absence of glucose (Supplementary Fig. 1a). In addition, AZD7762 increased luminescent signals from human EndoC-βH1 carrying proinsulin-NanoLuc reporter (NLuc-EndoC-βH1 cells) through a dose-dependent manner (Supplementary Fig. 1b). We further confirmed that AZD7762 stimulated insulin and C-peptide secretion from MIN6 cells and human EndoC-βH1 cells using ELISA. Consistent with the dose curve, AZD7762 significantly increased both insulin (Extended Data Fig. 1b) and C-peptide (Extended Data Fig. 1c) secretion in MIN6 cells in the presence of 20 mM d-glucose, but not in the absence of glucose. In addition, AZD7762 treatment significantly increased insulin (Extended Data Fig. 1d) and C-peptide (Extended Data Fig. 1e) secretion of human EndoC-βH1 cells at both 0.5 mM and 20 mM d-glucose conditions. AZD7762 also increases insulin secretion of EndoC-βH1 cells through a glucose-dose-dependent manner at 2 mM, 5 mM and 11 mM d-glucose (Extended Data Fig. 1f). Consistent with the observed activity of 10 µM AZD7762, a lower dose of 1 µM AZD7762 also significantly increased insulin secretion from MIN6 and EndoC-βH1 cells during GSIS (Extended Data Fig. 1g,d). Moreover, EndoC-βH1 cells treated with AZD7762 for 24 h also exhibit elevated insulin secretion (Extended Data Fig. 1h). To determine whether AZD7762 functions through insulin processing, we measured the total cellular insulin and proinsulin levels in EndoC-βH1 cells treated with 1 µM and 10 µM AZD7762 and did not detect any difference (Supplementary Fig. 1c), indicating that AZD7762 does not promote insulin secretion through enhanced proinsulin to insulin processing. Notably, AZD7762 did not increase β-cell death as evidenced by unchanged percentages of propidium iodide (PI)^+^ EndoC-βH1 cells after 1 h (Supplementary Fig. 1d) or 24 h (Supplementary Fig. 1e) of AZD7762 treatment. In addition, AZD treatment promotes insulin secretion in the absence of starvation (Extended Data Fig. 1i). To investigate whether AZD7762 increases GSIS by relieving cellular stress on β cells caused by starvation, we assessed stress markers, including phospho-eIF2α and phospho-CHEK2, by western blot and found that starvation in 0.5 mM glucose did not increase the phosphorylation of either CHEK2 or eIF2α (Supplementary Fig. 1f–i). Therefore, AZD7762 does not increase GSIS by relieving cellular stress caused by glucose deprivation. Together, these data show that AZD7762 treatment potentiates GSIS in both mouse and human β cell lines in the presence of glucose.

### AZD7762 increases insulin secretion in human islets

**Extended Data Fig. 2:**
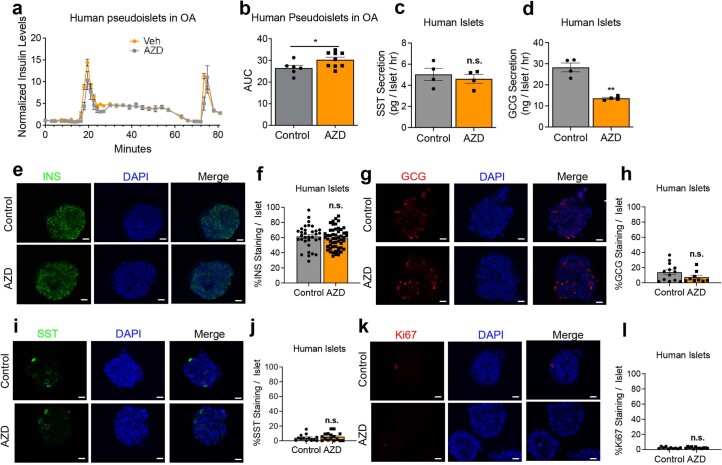
AZD7762 increases insulin secretion, but not GCG and SST from human islets. (**a** and **b**) Dynamic GSCS (**a**) and AUC (**b**) (*P*-value = 0.047) of human pseudoislets cultured in sodium oleate in the presence of control or 1 µM AZD7762. n = 6 (control) and n = 9 (AZD7762) biological replicates. The data was normalized to baseline. (**c**) SST secretion during GSIS of intact human islets in the presence of control or 1 µM AZD7762. n = 4 biological replicates. (**d**) glucagon secretion during GSIS of intact human islets in the presence of control or 1 µM AZD7762. *P*-value = 0.005. n = 4 biological replicates. (**e** and **f**) Immunostaining (**e**) and the quantification (**f**) of insulin staining in intact human islets treated with control or 1 µM AZD7762. n = 33 (control) and n = 53 (AZD7762) biological replicates. (**g** and **h**) Immunostaining (**g**) and the quantification (**h**) of glucagon staining in intact human islets treated with control or 1 µM AZD7762. n =13 (control) and n = 9 (AZD7762) biological replicates. (**i** and **j**) Immunostaining (**i**) and quantification (**j**) of SST staining in intact human islets treated with control or 1 µM AZD7762. n = 13 (control) and n = 16 (AZD7762) biological replicates. (**k** and **l**) Immunostaining (**k**) and the quantification (**l**) of Ki67 staining in intact human islets treated with control or 1 µM AZD7762. n = 10 (control) and n = 12 (AZD7762) biological replicates. For e, g, i and k, Scale bar: 100 μm. Data represents the Mean ± SEM. For b-d, f, h, j & l, *P*-value of figures were calculated by two-sided Student’s *t*-test. Statistical significance *p < 0.05, **p < 0.01, n.s. for not significantly different. Source data

We next tested if AZD7762 can improve insulin secretion from primary human islets. Consistent with the results using β cell lines, AZD7762-treated intact normal human islets (Supplementary Table 4) showed enhanced insulin secretion at both 2 mM and 20 mM d-glucose (Fig. 1c). We further validated the activity of AZD7762 on pseudoislets^17^, which showed a more robust and consistent response to glucose than intact islets. Each pseudoislet comprised approximately 2,000 dissociated human islet cells. Dynamic GSIS showed that AZD7762 treatment significantly increased secretion of insulin (Fig. 1d,e) and C-peptide (Fig. 1f,g), as well as the total area under the curve (AUC; Fig. 1e (insulin) and Fig. 1g (C-peptide)). To evaluate the activity of AZD7762 on pseudoislets that are similar to those in T2D condition, we cultured pseudoislets generated from healthy islets in 0.4 mM sodium oleate for 2 d to induce lipotoxicity and then assessed the short-term effect of AZD7762 on GSIS. One-hour treatment with AZD7762 significantly increased GSIS from pseudoislets cultured in lipotoxic condition (Extended Data Fig. 2a,b). The effect of AZD7762 was also confirmed using intact islets from T2D donors. Consistent with the impact on human EndoC-βH1 cells and healthy human islets, AZD7762 significantly improved GSIS (Fig. 1h) and glucose-stimulated C-peptide secretion (GSCS; Fig. 1i) in static condition. The perfusion experiments further validated that AZD7762 increased C-peptide secretion in response to 20 mM d-glucose (Fig. 1j). AUC was significantly increased in AZD7762-treated T2D islets (Fig. 1k). Then, we investigated if AZD7762 altered the secretion of other islet hormones and found that AZD7762 treatment did not alter secretion of SST from intact human islets (Extended Data Fig. 2c). AZD7762 treatment significantly reduced glucagon (GCG) secretion from intact normal human islets, possibly due to the inhibited effects of insulin on glucagon secretion, an observation reported by several groups^18,19^ (Extended Data Fig. 2d). Furthermore, immunostaining experiments confirmed that AZD7762 treatment does not change the percentages of insulin (INS)-, GCG-, SST- and Ki67-positive cells in human islets (Extended Data Fig. 2e–l). These results suggest that AZD7762 stimulates insulin secretion in primary human islets without changing the islet cellular composition.

### AZD7762 improves insulin secretion in mouse models

**Fig. 2:**
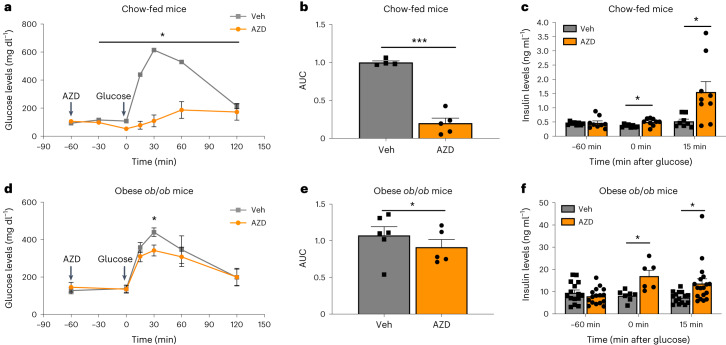
AZD7762 improves glucose tolerance and increases insulin secretion in healthy and T2D mouse models. **a**–**f**, Mice were fasted overnight before all GTT and GSIS experiments. **a**,**b**, IPGTT (**a**, *P* value: −30 min, *P* = 0.027; 0 min, *P* = 0.009; 15 min, *P* = 0.00003; 30 min, *P* = 0.0001; 60 min, *P* = 0.004) and AUC (**b**, *P* = 0.0001) of 8–12 weeks old male chow-fed CD-1/ICR mice treated with vehicle or 25 mg kg^−1^ AZD7762. *n* = 4 vehicle-treated mice; *n* = 5 AZD7762-treated mice. **c**, Intraperitoneal GSIS of chow-fed 8- to 12-week-old male CD-1/ICR mice treated with vehicle or 25 mg kg^−1^ AZD7762. *n* = 8 vehicle-treated mice; *n* = 9 AZD7762-treated mice (*P* value: 0 min, *P* = 0.043; 15 min, *P* = 0.022). **d**,**e**, IPGTT (**d**, *P* value: 30 min, *P* = 0.029) and AUC (**e**, *P* = 0.0296) of 16-week-old obese *ob*/*ob* mice treated with vehicle or 25 mg kg^−1^ AZD7762. *n* = 5 vehicle-treated mice; *n* = 6 AZD7762-treated mice. **f**, GSIS of 16-week-old *ob*/*ob* mice treated with vehicle or 25 mg kg^−1^ AZD7762. *P* value: 0 min, *P* = 0.016; 15 min, *P* = 0.01. For 0 min, *n* = 7 vehicle-treated mice, *n* = 6 AZD7762-treated mice. For 15 min, *n* = 15 vehicle-treated mice, *n* = 18 AZD7762-treated mice. For 60 min, *n* = 15 vehicle-treated mice, *n* = 16 AZD7762-treated mice. Data represent the mean ± s.e.m. *P* values of all figures were calculated by two-sided Student’s *t*-test. Statistical significance: *P < 0.05, ***P < 0.001. Source data

**Extended Data Fig. 3:**
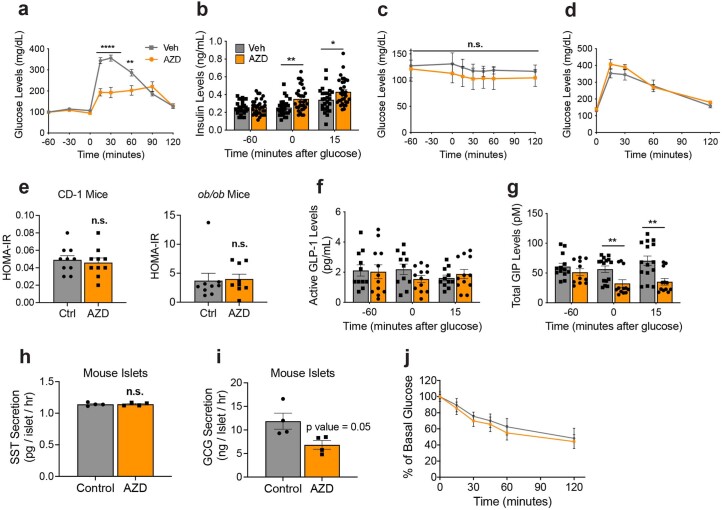
Low-dose AZD7762 does not cause hypoglycemia in mice. (**a**) IPGTT of 8–12 weeks old male chow-fed CD-1/ICR vehicle- or 12 mg/kg AZD7762-treated mice. *P*-value: 15 min, p = 1.00E-05; 30 min, p = 8.00E-06; 60 min, p = 0.006. n = 20 (vehicle); n = 14 (AZD7762). (**b**) GSIS of chow-fed 8–12 weeks old male CD-1/ICR vehicle- or 12 mg/kg AZD7762-treated mice. *P*-value: 0 min, p = 0.002; 15 min, p = 0.017. n = 25 (vehicle); n = 32 (AZD7762). (**c**) Glucose levels of chow-fed 8–12 weeks old male CD-1/ICR vehicle- or 12 mg/kg AZD7762-treated mice without glucose injection. n = 8 (vehicle); n = 7 (AZD7762). (**d**) IPGTT of 8–12 weeks old male chow-fed CD-1/ICR vehicle- or 12 mg/kg AZD7762-treated mice 24 hours before the experiment. n = 8 (vehicle); n = 7 (AZD7762). (**e**) HOMA-IR of vehicle- or 25 mg/kg AZD7762-treated mice. Left: 8–12 weeks old male chow-fed CD-1/ICR mice. n = 10 mice per group. Right: 16-week-old male obese *ob/ob* vehicle- or 25 mg/kg AZD7762-treated mice. n = 9 mice per group. (**f**) Active GLP-1 levels during IPGTT of chow-fed 8–12 weeks old male CD-1/ICR vehicle- or 12 mg/kg AZD7762-treated mice. n = 11(vehicle); n = 12 (AZD7762). (**g**) Total GIP levels during IPGTT of chow-fed 8–12 weeks old male CD-1/ICR vehicle- or 12 mg/kg AZD7762-treated mice. *P*-value: 0 min, p = 0.008; 15 min, p = 0.001. n = 15 (vehicle); n = 12 (AZD7762). (**h**) SST secretion during GSIS of intact mouse islets with control or 1 µM AZD7762. n = 4 biological replicates. (**i**) glucagon secretion during GSIS of intact mouse islets in the with control or 1 µM AZD7762. n = 4 biological replicates. (**j**) Glucose levels during insulin tolerance test of 8-month-old male chow-fed wildtype and *Chk2*^*−/−*^ mice. n = 4 (wildtype), n = 5 (*Chk2*^*−/−*^). Mice were fasted overnight for GTT and GSIS experiments. Data represent the Mean ± SEM. For a-e & h-i, *P*-value were calculated by two-sided Student’s *t*-test. Statistical significance *p < 0.05, **p < 0.01, n.s. for not significantly different. Source data

Because AZD7762 effectively increased insulin secretion from β cells in vitro, we further evaluated its impact on glucose metabolism in vivo using both healthy and T2D mouse models. In the glucose tolerance test (GTT), overnight-fasted chow-fed CD-1/ICR mice were first intraperitoneally (IP) treated with 25 mg kg^−1^ of AZD7762. One hour later, mice were injected IP with glucose for GTT and GSIS experiments. AZD7762 significantly improved glucose tolerance in chow-fed CD-1/ICR mice (Fig. 2a,b). Consistently, AZD7762-treated mice show significantly increased insulin secretion compared to vehicle-treated mice at 0- and 15-min postglucose injection (Fig. 2c). Because 25 mg kg^−1^ AZD7762 led to a slight reduction in glucose levels in mice before glucose administration, we evaluated the effect of lower dose of AZD7762 on glucose tolerance and GSIS. At a dose of 12 mg kg^−1^, AZD7762 treatment did not cause hypoglycemia at 0 min, while still significantly improving glucose tolerance and insulin secretion at both 0- and 15-min postglucose injection (Extended Data Fig. 3a,b). Notably, even when no glucose was administered for a total period of 3 h, treatment with 12 mg kg^−1^ AZD7762 did not result in hypoglycemia (Extended Data Fig. 3c). The effect of AZD7762 on glucose tolerance is short-term as there was no change in glucose tolerance in mice 24 h after AZD7762 treatment (Extended Data Fig. 3d). This result is consistent with the half-life of AZD7762 reported to be only 1–2 h in mice^20^. Next, two T2D mouse models, HFD-fed C57BL/6J mice and genetically obese leptin-deficient *Lep*^*ob*^^/^^*ob*^ (hereafter *ob*/*ob* mice) mice were used to evaluate the effect of AZD7762. Similar to the effect on chow-fed mice, 1-h pretreatment with AZD7762 significantly improved glucose tolerance during intraperitoneal glucose tolerance test (IPGTT) in C57BL/6J mice fed with HFD for 4 months (Supplementary Fig. 2a,b). Insulin secretion was also higher in AZD7762-treated mice, but the increase in insulin secretion only reached significance at 30 min postglucose administration (Supplementary Fig. 2c). Additionally, 16-week-old *ob*/*ob* mice treated with AZD7762 showed improved glucose tolerance (Fig. 2d,e) and enhanced insulin secretion at 15 min postglucose administration compared with vehicle-treated *ob*/*ob* mice (Fig. 2f). We calculated the homeostasis model assessment of insulin resistance (HOMA-IR) in AZD-treated and control chow-fed CD-1/ICR, HFD-fed C57BL/6J and *ob*/*ob* mice, and found no significant differences between the vehicle and AZD7762 treatment groups (Extended Data Fig. 3e and Supplementary Fig. 2d).

To determine the possible effect of AZD7762 on incretin hormones, we measured active GLP-1 and total GIP levels during in vivo GSIS of CD-1 mice at 0- and 15-min postglucose injection. We did not detect a difference in GLP-1 levels between AZD7762- and vehicle-treated mice (Extended Data Fig. 3f). We observed a reduction in GIP levels at 0- and 15-min postglucose injection in AZD7762 mice treated with AZD7762 (Extended Data Fig. 3g), which is consistent with increased insulin levels as it was reported that insulin can inhibit GIP secretion in vivo^21–24^. Furthermore, we investigated if AZD7762 increases secretion of other islet hormones from primary mouse islets. Similar to primary human islets (Extended Data Fig. 2c,d), mouse islets treated with AZD7762 did not show any change in SST secretion but revealed insignificant reductions in glucagon secretion (Extended Data Fig. 3h,i). Furthermore, AZD7762-treated mice did not show an improvement in insulin sensitivity during an insulin tolerance test (Extended Data Fig. 3j). Together, the in vivo data strongly suggest that AZD7762 improves glucose homeostasis by stimulating insulin secretion in β cells without altering insulin sensitivity.

### AZD7762 enhances GSIS in cynomolgus macaques

**Fig. 3:**
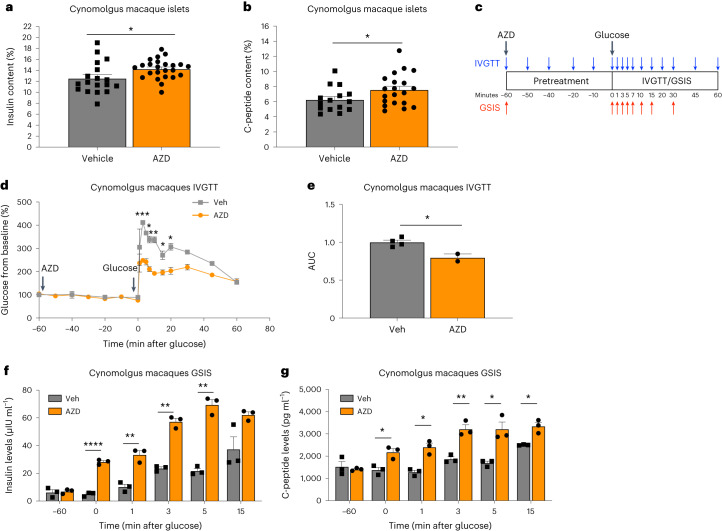
AZD7762 enhances glucose tolerance and GSIS in cynomolgus macaques. **a**, Insulin secretion of cynomolgus macaque islets with 20 mM glucose in the presence of control or 1 µM AZD7762. *P* = 0.032. *n* = 18 (control) and *n* = 24 (AZD7762) biological replicates. **b**, C-peptide secretion of cynomolgus macaque islets with 20 mM glucose in the presence of control or 1 µM AZD7762. *P* = 0.046. *n* = 15 (control) and *n* = 20 (AZD7762) biological replicates. **c**, Schematics of IVGTT and GSIS experiments. **d**,**e**, Glucose levels during IVGTT (**d**, *P* value: 3 min, *P* = 0.0001; 5 min, *P* = 0.014; 7 min, *P* = 0.001; 10 min, *P* = 0.001; 15 min, *P* = 0.025; 20 min, *P* = 0.015) and AUC (**e**, *P* = 0.018) in male cynomolgus macaques (11 and 13 years old, respectively) pretreated with control vehicle or 1.6 mg kg^−1^ AZD7762. Vehicle-treated animals (*n* = 4 biological replicates), AZD7762-treated animals (*n* = 2 biological replicates). **f**, Insulin secretion during IV glucose infusion in cynomolgus macaques pretreated with vehicle or 1.6 mg kg^−1^ AZD7762. *P* value: 0 min, *P* = 0.00005; 1 min, *P* = 0.004; 3 min, *P* = 0.001; 5 min, *P* = 0.002. Vehicle-treated animals (*n* = 3 biological replicates), AZD7762-treated animals (*n* = 3 biological replicates). **g**, C-peptide secretion during IV glucose infusion in cynomolgus macaques receiving control vehicle or 1.6 mg kg^−1^ AZD7762. *P* value: 0 min, *P* = 0.018; 1 min, *P* = 0.011; 3 min, *P* = 0.008; 5 min, *P* = 0.032; 15 min, *P* = 0.037. Vehicle-treated animals (*n* = 3 biological replicates), AZD7762-treated animals (*n* = 3 biological replicates). Data represent the mean ± s.e.m. *P* value was calculated with two-sided Student’s *t*-test for all figures. Statistical significance: *P < 0.05, **P < 0.01, ***P < 0.001, ****P < 0.0001. Source data

We further evaluated AZD7762’s activity using cynomolgus macaques, a nonhuman primate model. AZD7762-treated cynomolgus macaque islets showed a significant increase in insulin and C-peptide secretion when stimulated with 20 mM d-glucose (Fig. 3a,b). To determine the in vivo activity of AZD7762 on cynomolgus macaques, we performed an intravenous glucose tolerance test (IVGTT). The animals were injected with 1.6 mg kg^−1^ of AZD7762 1 h before the experiment (Fig. 3c). Pretreatment with AZD7762 significantly increased glucose tolerance (Fig. 3d,e) with approximately 20% reduction in AUC. The improvement in glucose tolerance was accompanied by a significant increase in insulin secretion (Fig. 3f) and C-peptide secretion (Fig. 3g) at 0-, 1, 3-, 5- and 15-min post-IV-glucose injection. These results show that AZD7762 does not only improve GSIS in primary human islets in vitro but also improves GSIS and glucose homeostasis in cynomolgus macaques in vivo, supporting a possible physiological role of CHEK2 in modulating insulin secretion in response to glucose in primates.

### Reduced CHEK2 in human β cells improves insulin secretion

**Fig. 4:**
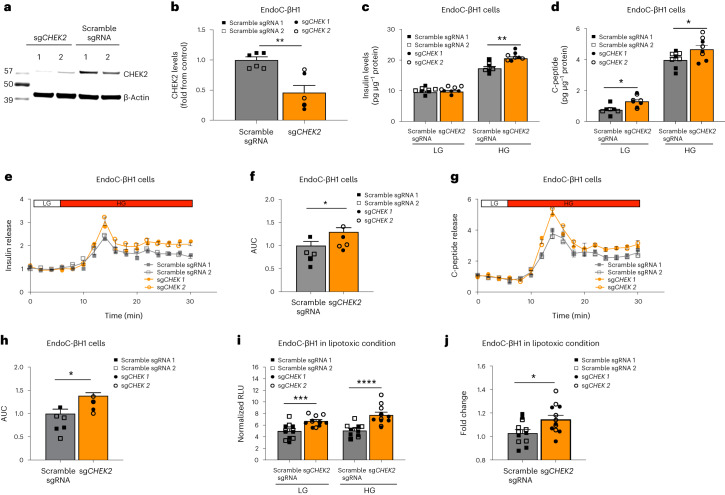
Reduction of CHEK2 in human β cells increases insulin secretion. **a**,**b**, Western blotting (**a**) and quantification (**b**) of CHEK2 in EndoC-βH1 cells carrying scrambled sgRNA or sg*CHEK2*. *P* = 0.0031. *n* = 6 biological replicates per group. **c**, GSIS of EndoC-βH1 cells carrying scrambled sgRNA or sg*CHEK2*. LG, 0.5 mM; HG, 20 mM (*P* = 0.0014). *n* = 8 biological replicates for each group. **d**, GSCS of EndoC-βH1 cells carrying scrambled sgRNA or sg*CHEK2*. LG, 0.5 mM (*P* = 0.0106); HG, 20 mM (*P* = 0.029). For LG, *n* = 7 (scrambled sgRNA) and *n* = 8 (sg*CHEK2*) biological replicates. For HG, *n* = 8 biological replicates per group. **e**,**f**, Dynamic GSIS (**e**) and AUC (**f**, *P* = 0.0116) of EndoC-βH1 pseudoislets carrying scrambled sgRNA or sg*CHEK2*. LG: 0.5 mM; HG: 20 mM. *n* = 5 biological replicates per group. The data were normalized to baseline. **g**,**h**, Dynamic GSCS (**g**) and AUC (**h**, *P* = 0.0277) of EndoC-βH1 pseudoislets carrying scrambled sgRNA or sg*CHEK2*. LG: 0.5 mM; HG: 20 mM. *n* = 5 biological replicates per group. The data were normalized to baseline. **i**,**j**, Nanoluciferase secretion (**i**, *P* value: LG, 0.0009; HG, 0.00005) and fold change (**j**, *P* = 0.0197) during GSIS of EndoC-βH1 nanoluciferase reporter cells carrying scrambled sgRNA or sg*CHEK2* cultured in 2 mM sodium oleate. *n* = 12 biological replicates per group. Data represent the mean ± s.e.m. *P* values were calculated by mixed ANOVA. Statistical significance: *P < 0.05, **P < 0.01, ***P < 0.001, ****P < 0.0001. RLU is used as an acronym for Relative Light Unit. Source data

**Extended Data Fig. 4:**
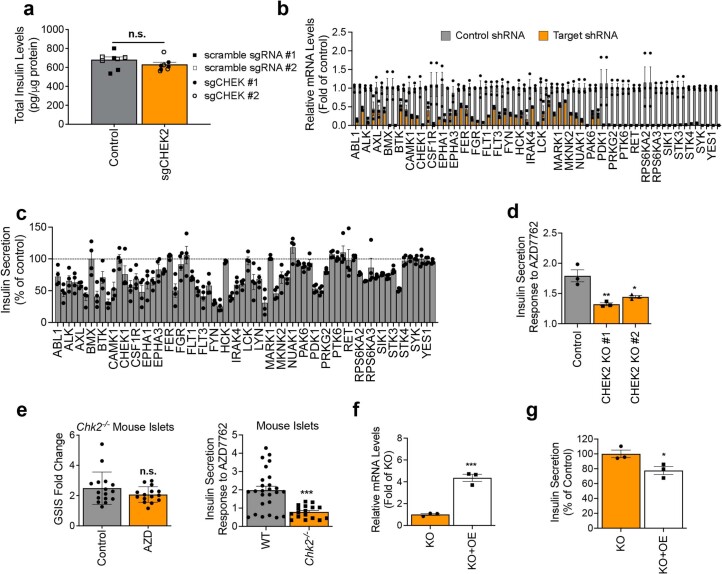
CHEK2 mediates the effect of AZD7762 on insulin secretion. (**a**) Total insulin levels in EndoC-βH1 cells carrying scramble sgRNA or sgCHEK2. n = 8 biological replicates. (**b**) Quantitative RT-PCR for gene expression of AZD7762 off-target genes knockdown by shRNA in EndoC-βH1 cells. n = 3 biological replicates. Gene expression was normalized to that of b-Actin and presented as fold changes (± SEM) against control expression. (**c**) NLuc secretion of NLuc-EndoC-βH1 cells carrying shRNA targeting AZD7762 off-targets during GSIS. n = 3 biological replicates. (**d**) Insulin secretion response to AZD7762 in control or sgCHEK2 EndoC-βH1 cells. *P*-value: Control vs CHEK2 KO #1, p = 0.003; Control vs CHEK2 KO #2, p = 0.012. n = 3 biological replicates. (**e**) Left: GSIS of *Chk2*^*−/−*^ mouse islets in the presence of control or 1 µM AZD7762. n = 16 (control) and n = 15 (AZD7762) biological replicates. Right: Insulin secretion response to AZD7762 in wildtype or *Chk2*^*−/−*^ mouse islets in the presence of control or 1 µM AZD7762. *P*-value = 0.00001. n = 26 (wildtype) and n = 17 (*Chk2*^*−/−*^) biological replicates. (**f**) Quantitative RT-PCR for *CHEK2* expression in control (KO) and CHEK2 KO-overexpressed (KO+OE) EndoC-βH1 cells. *P*-value = 0.000597. n = 3 biological replicates. Gene expression was normalized to that of b-Actin and presented as fold changes (± SEM) against control expression. (**g**) GSIS of control (KO) and CHEK2 KO-overexpressed (KO+OE) EndoC-βH1 cells. *P*-value = 0.04. n = 3 biological replicates. Data represent the Mean ± SEM. For a, *P*-values of figures were calculated mixed ANOVA. For b-c, *P*-values of figures were calculated by one-sided Student’s *t*-test. For d, *P*-value of figures were calculated by one-way ANOVA (Dunnett′s test). For e-g, *P*-value of figures were calculated by two-sided Student’s *t*-test. Statistical significance *p < 0.05, **p < 0.01, ***p < 0.001, n.s. for not significantly different. Source data

Because both AZD7762 (a competitive CHEK1/CHEK2 inhibitor)^15^ and CCT241533 (2, a selective CHEK2 inhibitor)^25^ (Supplementary Table 3) were identified to potentiate GSIS in the primary screen, we decided to apply genetic approaches to confirm and determine the role of *CHEK2* in β-cell function. We generated *CHEK*2-deficient EndoC-βH1 (hereafter known as sg*CHEK2* EndoC-βH1) cells by using a CRISPR-Cas9-mediated knockout approach. In brief, EndoC-βH1 cells were infected with a lentivirus carrying single guide RNA (sgRNA) against exon 2 of *CHEK2* (sg*CHEK2*) or a scrambled control sgRNA (Supplementary Table 5). After 1 week of puromycin selection, western blotting confirmed a significant reduction in CHEK2 protein levels (Fig. 4a,b). No detectable difference in total insulin was observed between EndoC-βH1 cells carrying control scrambled sgRNA or sg*CHEK2* (Extended Data Fig. 4a), suggesting that CHEK2 is not involved in insulin synthesis. Static GSIS confirmed that EndoC-βH1 cells carrying sg*CHEK2* showed an increase in insulin (Fig. 4c) and C-peptide (Fig. 4d) secretion when stimulated with 20 mM d-glucose, suggesting that the reduction of CHEK2 led to an increased response to glucose stimulation. EndoC-βH1 cells carrying scrambled sgRNA or sg*CHEK2* were then aggregated to form pseudoislets and used for dynamic GSIS. Consistent with the static GSIS results, the pseudoislets containing EndoC-βH1 cells carrying sg*CHEK2* showed enhanced insulin response (Fig. 4e) and AUC (Fig. 4f), as well as C-peptide secretion (Fig. 4g,h). To further examine the impact of the loss of CHEK2 on β-cell function in T2D condition, NLuc-EndoC-βH1 cells carrying control scrambled sgRNA or sg*CHEK2* were cultured in the presence of 2 mM sodium oleate for 72 h and then assessed for their insulin secretory response to glucose. Similar to other reports on β-cell dysfunction induced by lipotoxicity, NLuc-EndoC-βH1 cells carrying scrambled sgRNA cultured in sodium oleate showed diminished insulin secretion in response to glucose stimulation (Fig. 4i), while NLuc-EndoC-βH1 cells carrying sg*CHEK2* showed higher insulin response to glucose stimulation than the control (Fig. 4i,j). These data suggest that the pathway that mediates CHEK2’s effect on GSIS is at least partially preserved in lipotoxic T2D-like condition.

Considering that AZD7762 is known to target other kinases, we conducted a shRNA knockdown experiment to evaluate the effect of AZD7762’s off-targets on GSIS. We first performed a kinase screening assay using AZD7762 and observed that 36 of the 37 reported kinase targets of AZD7762 (ref. ^15^), including CHEK2, were inhibited by more than 40% (Supplementary Tables 6 and 7). Subsequently, we designed two shRNAs to knockdown each of the off-target genes in EndoC-βH1 cells (Supplementary Table 8) and evaluated the effects of these gene knockdowns on GSIS (Extended Data Fig. 4b). Notably, knockdown of any of the off-target genes of AZD7762, including *CHEK1*, did not result in an increase in GSIS (Extended Data Fig. 4c), further confirming that AZD7762 primarily exerts its effects through *CHEK2*. We also evaluated the impact of AZD7762 on shCHEK2 EndoC-βH1 cells and *Chk2*^*−*/*−*^ mouse islets and found that the GSIS response to AZD7762 treatment was significantly reduced in CHEK2-deficient β cells (Extended Data Fig. 4d,e). Consistent with the impact of CHEK2 deficiency on GSIS, overexpression of *CHEK2* suppressed insulin secretion from shCHEK2 EndoC-βH1 cells (Extended Data Fig. 4f,g). Overall, these results confirm that the effect of AZD7762 on GSIS is mediated via suppressing CHEK2 functions, and suppressing CHEK2 functions can improve insulin secretion in both normal and related conditions in vitro.

### *Chk2*^*−*/*−*^ mice show improved GSIS in HFD condition

**Fig. 5:**
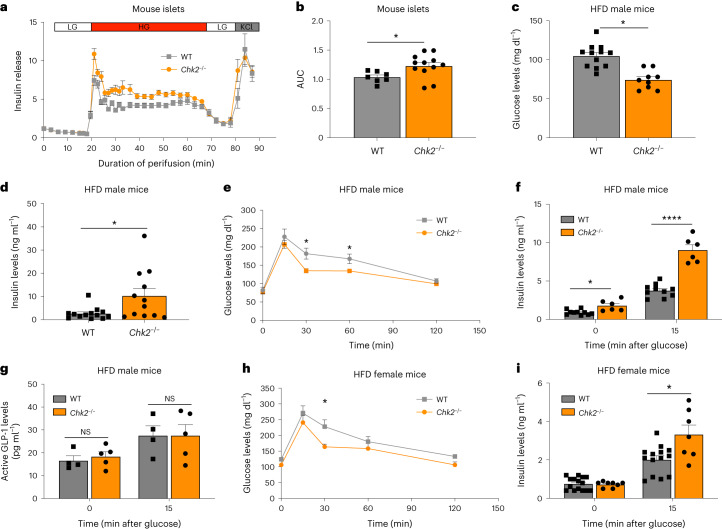
HFD *Chk2*^*−*/*−*^ mice show improved GSIS and glucose homeostasis. **a**,**b**, Dynamic GSIS (**a**) and AUC (**b**, *P* = 0.012) of primary mouse islets from chow-fed wild-type and *Chk2*^*−*/*−*^ mice. *n* = 7 wild-type mice; *n* = 12 *Chk2*^*−*/*−*^ mice. The data were normalized to baseline. **c**, Fed glucose levels in 8-month-old male HFD-fed wild-type and *Chk2*^*−*/*−*^ mice. *P* = 0.00005. *n* = 12 wild-type mice; *n* = 9 *Chk2*^*−*/*−*^ mice. **d**, Fed insulin levels of 8-month-old male HFD-fed wild-type and *Chk2*^*−*/*−*^ mice. *P* = 0.034. *n* = 13 wild-type mice; *n* = 12 *Chk2*^*−*/*−*^ mice. **e**, OGTT of 8-month-old male HFD-fed wild-type and *Chk2*^*−*/*−*^ mice. *P* value: 30 min, *P* = 0.0138; 60 min, *P* = 0.036. *n* = 11 wild-type mice; *n* = 8 *Chk2*^*−*/*−*^ mice. **f**, Insulin levels during OGTT of 8-month-old male HFD-fed wild-type and *Chk2*^*−*/*−*^ mice. *P* value: 0 min, *P* = 0.026; 15 min, *P* = 0.0002. *n* = 10 wild-type mice, *n* = 6 *Chk2*^*−*/*−*^ mice. **g**, Active GLP-1 levels during OGTT of 8-month-old male HFD-fed wild-type and *Chk2*^*−*/*−*^ mice. *n* = 4 wild-type mice; *n* = 5 *Chk2*^*−*/*−*^ mice. **h**, Glucose levels during OGTT of 8-month-old female HFD-fed wild-type and *Chk2*^*−*/*−*^ mice. *n* = 5 wild-type mice; *n* = 3 *Chk2*^*−*/*−*^ mice. **i**, Insulin levels during OGTT of 8-month-old female HFD-fed wild-type and *Chk2*^*−*/*−*^ mice. *P* = 0.036. *n* = 15 wild-type mice; *n* = 8 *Chk2*^*−*/*−*^ mice. Mice were fasted overnight for all GTT and GSIS experiments. Data represent the mean ± s.e.m. For **b**–**i**, *P* value was calculated with two-sided Student’s *t*-test. Statistical significance: *P < 0.05, ****P < 0.0001. NS, not significantly different, WT, wild type. Source data

To confirm the physiological relevance of the loss of *CHEK2* (*Chk2* in mouse) in vivo, we characterized the glucose metabolism profile in *Chk2*^*−*/*−*^ mice^26–28^. We first isolated islets from chow-fed *Chk2*^*−*/*−*^ mice for insulin secretion assays to directly assess β-cell function. In vitro dynamic GSIS assay showed that chow-fed *Chk2*^*−*/*−*^ mouse islets had increased insulin secretion in response to 20 mM d-glucose stimulation (Fig. 5a,b). However, adult *Chk2*^*−*/*−*^ mice on a chow diet did not show any detectable difference in whole-body basal glucose and insulin levels (Supplementary Fig. 3a,b), glucose tolerance (Supplementary Fig. 3c), insulin secretion during GSIS (Supplementary Fig. 3d) and insulin tolerance (Extended Data Fig. 3j) when compared to wild-type mice.

**Extended Data Fig. 5:**
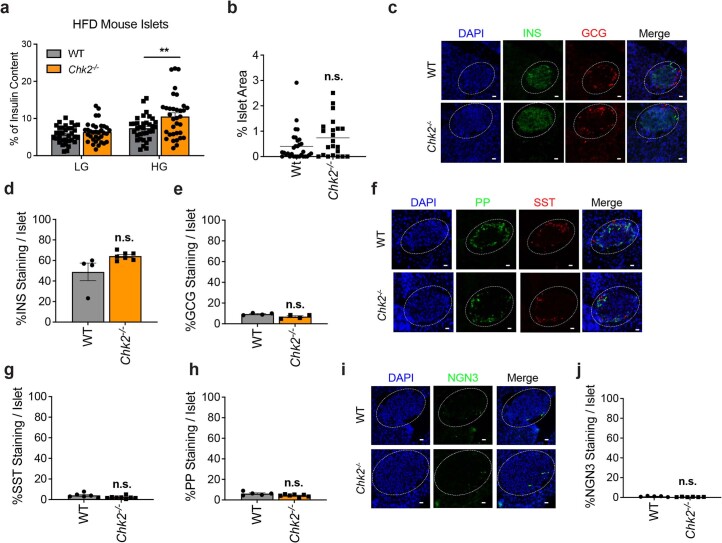
HFD Chk2−/− mice do not exhibit changes in insulin sensitivity or islet cellular composition and histology. (**a**) Static GSIS of pancreatic islets isolated from 8-month-old HFD-fed wildtype and *Chk2*^*−/−*^ mice. LG: 2 mM; HG: 20 mM. *P*-value = 0.008. n = 31 wildtype mice; n = 22 *Chk2*^*−/−*^ mice. (**b**) The percent of islet area per pancreatic section in 8-month-old male HFD-fed wildtype and *Chk2*^*−/−*^ mice. n = 31 wildtype mice; n = 22 *Chk2*^*−/−*^ mice. (**c, d** and **e**) Immunostaining (c) and the quantification of insulin (d) and glucagon (e) staining in pancreatic islets (outlined by dotted line) of 8-month-old male HFD-fed wildtype and *Chk2*^*−/−*^ mice. For (**d**), n = 4 wildtype mice; n = 7 *Chk2*^*−/−*^ mice per group. For (**e**), n = 4 wildtype mice; n = 4 *Chk2*^*−/−*^ mice per group. Arrows indicate the localization of cells expressing respective markers: α-cells (red) and β-cells (green). (**f, g** and **h**) Immunostaining (**f**) and the quantification of SST (**g**) and PP (h) staining in pancreatic islets (outlined by dotted line) of 8-month-old male HFD-fed wildtype and *Chk2*^*−/−*^ mice. For (**g**), n = 6 wildtype mice; n = 8 *Chk2*^*−/−*^ mice. For (**h**), n = 5 wildtype mice; n = 7 *Chk2*^*−/−*^ mice. Arrows indicate the localization of cells expressing respective markers: δ-cells (red) and γ-cells (green). (**i** and **j**) Immunostaining (**i**) and the quantification of Ngn3 (**j**) staining in pancreatic islets (outlined by dotted line) of 8-month-old male HFD-fed wildtype and *Chk2*^*−/−*^ mice. n = 5 wildtype mice; n = 6 *Chk2*^*−/−*^ mice. Arrows indicate the localization of cells expressing NGN3 (green). For c, f and i, Scale bar: 100 μm. Data represents the Mean ± SEM. All *P*-value was calculated with two-sided Student’s *t*-test. Statistical significance **p < 0.01, n.s. for not significantly different. Source data

Our previous data showed that the reduction of CHEK2 improved human β-cell function in T2D conditions in vitro (Fig. 4i,j). We therefore evaluated the glucose metabolism in *Chk2*^*−*/*−*^ mice fed with 60% HFD for 6 months. Male *Chk2*^*−*/*−*^ mice exhibited decreased fed glucose levels (Fig. 5c) and increased fed insulin levels (Fig. 5d). Moreover, HFD-fed male *Chk2*^*−*/*−*^ mice displayed significant improvement in oral glucose tolerance test (OGTT; Fig. 5e), accompanied by an increase in insulin secretory response after glucose administration (Fig. 5f). Similar to AZD7762-treated mice (Extended Data Fig. 3f), active GLP-1 levels were not significantly different between wild-type and *Chk2*^*−*/*−*^ mice, suggesting that the increased insulin secretion during OGTT was not due to changes in active GLP-1 levels (Fig. 5g). Similar to the male *Chk2*^*−*/*−*^ mice, HFD-fed female *Chk2*^*−*/*−*^ mice also showed improved glucose tolerance (Fig. 5h) and increased insulin secretion (Fig. 5i) during OGTT. We further isolated islets from HFD-fed *Chk2*^*−*/*−*^ mice to confirm the increase in insulin secretion in vitro. HFD *Chk2*^*−*/*−*^ mouse islets showed enhanced insulin secretion in response to 20 mM d-glucose when compared to HFD-fed wild-type mouse islets (Extended Data Fig. 5a). Together, these results show that the loss of CHEK2 improves GSIS in normal and T2D conditions and *Chk2*^*−*/*−*^ mice exhibit increased resistance to HFD-induced β-cell dysfunction.

To further elucidate the effect of *Chk2* loss-of-function in vivo in islets, we performed immunostaining of islet cell markers on pancreatic sections from HFD-fed wild-type and *Chk2*^*−*/*−*^ mice to determine if *Chk2* loss-of-function led to any changes in the cellular composition of the pancreatic islet (Extended Data Fig. 5b). Immunostaining of INS, GCG, SST and PP did not uncover any changes in percentage islet area or islet cell markers in HFD-fed *Chk2*^*−*/*−*^ mice (Extended Data Fig. 5c–h). Consistent with unchanged percentage of INS-positive cells in HFD-fed *Chk2*^*−*/*−*^ mouse islets, the percentage of cells expressing neurogenin-3 (NGN3), a dedifferentiation marker of β cell^29^, in pancreatic islets was also not changed in HFD-fed *Chk2*^*−*/*−*^ mice (Extended Data Fig. 5i,j). Hematoxylin and eosin (H&E) staining of pancreatic section also did not reveal any changes in HFD-fed *Chk2*^*−*/*−*^ mouse islets (Supplementary Fig. 3e). Together, these data suggest that loss of CHEK*2* does not change the islet structure or cellular composition.

### Metabolomics reveal CHEK2–PP2A–PLK1–G6PD–PPP pathway

**Fig. 6:**
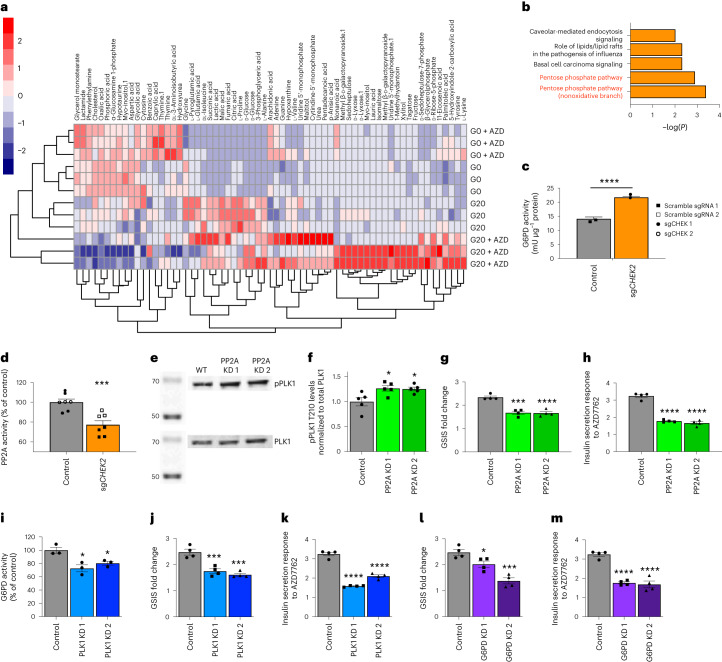
Untargeted metabolomics identified that a CHEK2/pentose phosphate axis is involved in insulin secretion. **a**, Heatmap of hits with VIP > 1 in untargeted metabolomics profiles. **b**, IPA analysis of the untargeted metabolomics profiles identified top pathways altered in 10 µM AZD7762-treated MIN6 cells in the presence of 20 mM glucose. **c**, G6PD activity of EndoC-βH1 cells carrying control or sg*CHEK2* stimulated with 20 mM glucose. *P* = 0.00008. *n* = 4 biological replicates. **d**, PP2A activity of EndoC-βH1 cells carrying control or sg*CHEK2* stimulated with 20 mM glucose. *P* = 0.0003. *n* = 7 biological replicates per group. **e**,**f**, Western blotting (**e**) and quantification (**f**) of phospho-PLK1 Thr210 in control and sh*PP2A* EndoC-βH1 cells. *P* = 0.02 between control and PP2A KD1. *P* = 0.025 between control and PP2A KD2. *n* = 4 biological replicates per group. **g**, GSIS of control and sh*PP2A* EndoC-βH1 cells in the presence of 10 µM AZD7762. *P* = 1.01 × 10^−4^ between control and PP2A KD (sh*PP2A*) 1. *P* = 8.69 × 10^−5^ between control and PP2A KD (sh*PP2A*) 2. *n* = 4 biological replicates. **h**, Insulin secretion response to AZD7762 of control and sh*PP2A* EndoC-βH1 cells. *P* = 1.82 × 10^−6^ between control and PP2A KD (sh*PP2A*) 1. *P* = 7.16 × 10^−8^ between control and PP2A KD (sh*PP2A*) 2. *n* = 4 biological replicates. **i**, G6PD act**i**vity of control and sh*PLK1* EndoC-βH1 cells. *P* = 0.0081 between control and PLK1 KD (sh*PLK1)* 1. *P* = 0.035 between control and PLK1 KD (sh*PLK1*) 2. *n* = 3 biological replicates. **j**, GSIS of control and sh*PLK1* EndoC-βH1 cells. *P* = 0.0005 between control and PLK1 KD (sh*PLK1*) 1. *P* = 0.0001 between control and PLK1 KD (sh*PLK1*) 2. *n* = 4 biological replicates. **k**, Insulin secretion response to AZD7762 of control and sh*PLK1* EndoC-βH1 cells. *P* = 7.16 × 10^−8^ between control and PLK1 KD (sh*PLK1*) 1. *P* = 1.82 × 10^−6^ between control and PLK1 KD (sh*PLK1*) 2. *n* = 4 biological replicates. **l**, GSIS of control and sh*G6PD* EndoC-βH1 cells. *P* = 0.041 between control and G6PD KD (sh*G6PD*) 1. *P* = 0.0002 between control and G6PD KD (sh*G6PD*) 2. *n* = 4 biological replicates. **m**, Insulin secretion response to AZD7762 of control and sh*G6PD* EndoC-βH1 cells. *P* = 2.19 × 10^−5^ between control and G6PD KD (sh*G6PD*) 1. *P* = 1.42 × 10^−5^ between control and G6PD KD (sh*G6PD*) 2. *n* = 4 biological replicates. Data represent the mean ± s.e.m. For **c** and **d**, *P* value was calculated by mixed-model ANOVA. For **f**–**m**, *P* value was calculated by one-way ANOVA (Dunnett’s test). Statistical significance *P < 0.05, ***P < 0.001, ****P < 0.0001. Source data

**Extended Data Fig. 6:**
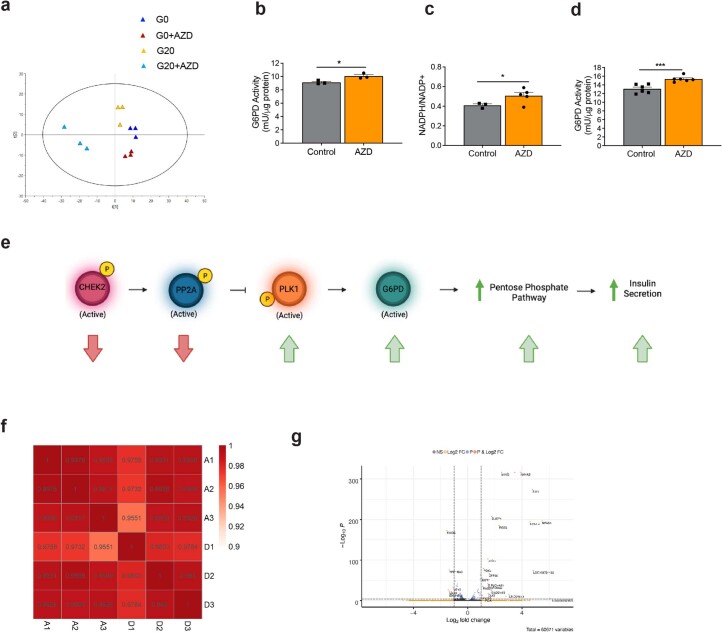
The CHEK2-PP2A-PLK1-G6PD-PPP pathway modulates insulin secretion in β cells. (**a**) PLS-DA scores plot of metabolomic profiles of MIN6 cells. MIN6 cells were starved in 0 mM glucose for 1 hour, and then in 0 mM glucose for an additional 1 hour, then stimulated with either 0 mM or 20 mM glucose for 30 minutes in the presence of 0 or 10 µM of AZD7762 throughout the experiment. (6 components, R2Xcum = 0.919, R2Ycum = 0.987, Q2cum=0.882). (**b**) G6PD activity in MIN6 cells in the presence of control or 10 µM AZD7762. n = 3 biological replicates. *P*-value = 0.034. (**c**) Cytosolic NADPH/NADP levels of MIN6 cells in the presence of control or 10 µM AZD7762. *P*-value = 0.04. n = 3 (control) and n = 5 (AZD7762) biological replicates. (**d**) G6PD activity in EndoC-βH1 cells treated with 10 µM AZD7762. *P*-value = 0.000994. n = 6 biological replicates. (**e**) Schematic representation of the CHEK2-PP2A-PLK1-G6PD-PPP pathway in insulin secretion. (**f**) Correlation matrix for bulk RNA-seq on control and 10 µM AZD-treated EndoC-βH1 cells. Control: D1-D3. AZD: A1-A3. (**g**) Volcano plot of differentially expressed genes for bulk RNA-seq on control and 10 µM AZD-treated EndoC-βH1 cells. For Extended For b-d, *P*-value was calculated by two-sided Student’s *t*-test. Data represent the Mean ± SEM. Statistical significance *p < 0.05, ***p < 0.001. Source data

To understand how reduced CHEK2 function potentiates insulin secretion, we performed an untargeted metabolomics profiling using MIN6 cells treated with control or AZD7762 in the absence or presence of 20 mM d-glucose. Partial least square-discriminant analysis (PLS-DA) scores plot confirmed that four different treatment groups clustered separately (Extended Data Fig. 6a). The heatmap highlighted a group of metabolites that were increased in AZD7762-treated cells only in the presence of 20 mM d-glucose (Fig. 6a). Metabolites reported to be associated with insulin secretion such as fructose, sedoheptulose and ribose-5-phosphate^30^ were only increased by AZD7762 treatment at 20 mM d-glucose, consistent with our observation that AZD7762 augmented insulin secretion at 20 mM d-glucose, but not at 0 mM glucose (Extended Data Fig. 1i). QIAGEN Ingenuity Pathway Analysis (IPA) analysis of these metabolites identified that PPP was significantly increased in AZD7762-treated cells upon 20 mM d-glucose stimulation (Fig. 6b).

To confirm the changes in PPP in AZD7762-treated cells upon 20 mM d-glucose stimulation, we assessed the activity of G6PD, the rate-limiting enzyme of PPP^31^. AZD7762 significantly increased G6PD activities (Extended Data Fig. 6b). Consistent with the changes in PPP and G6PD activity, cytosolic NADPH/NADP ratio was also significantly elevated in the presence of AZD7762 (Extended Data Fig. 6c). These data showed that acute pharmacological inhibition of CHEK2 activated PPP and increased G6PD activities, and cytosolic NADPH/NADP ratio. Consistently, AZD7762 significantly stimulated G6PD activities in EndoC-βH1 cells (Extended Data Fig. 6d). We then confirmed the change of PPP in genetically modified EndoC-βH1 cells. Similar to the results of pharmacological inhibition of CHEK2 with AZD7762, sgCHEK2 EndoC-βH1 cells also showed higher G6PD activity when compared to control EndoC-βH1 cells stimulated with 20 mM glucose (Fig. 6c). Together, our data show that potentiation of GSIS by CHEK2 inhibition requires activation of PPP and G6PD activities.

Given the absence of previous reports on a direct interaction between CHEK2 and G6PD, we hypothesize that other effectors might be involved in mediating the effect of CHEK2 on G6PD activity. Studies have demonstrated that CHEK2 binds and activates PP2A^32^, which in turn can deactivate PLK1 through dephosphorylation^33^. PLK1 has been shown to directly activate G6PD^34^, the rate-limiting enzyme for the PPP. We then used a shRNA knockdown strategy to explore the relationship between CHEK2, PP2A, PLK1 and G6PD, as well as to evaluate their contribution to regulating insulin secretion in β cells.

First, we demonstrated that *sgCHEK2* EndoC-βH1 cells exhibit a reduction in PP2A activity (Fig. 6d), which aligns with a previous report that CHEK2 phosphorylated and increased catalytic activity of PP2A (ref. ^32^). Subsequently, we evaluated the effect of *PP2A* loss-of-function in β cells by knocking down PP2A expression in EndoC-βH1 cells using a shRNA strategy (Supplementary Fig. 4a). We refer to these cells as sh*PP2A* EndoC-βH1 cells. Consistent with the previous report^33^, activation phosphorylation (T210) status of PLK1 was increased in sh*PP2A* EndoC-βH1 cells (Fig. 6e,f). sh*PP2A* EndoC-βH1 cells exhibited a reduction in AZD7762-potentiated GSIS (Fig. 6g,h). This is consistent with the role of PP2A in mediating CHEK2’s effect on insulin secretion. Next, we evaluated the function of PLK1 in β cells by infecting EndoC-βH1 cells with lentivirus carrying shRNA against *PLK1* (hereafter known as sh*PLK1* EndoC-βH1 cells; Supplementary Fig. 4b). Reduction of *PLK1* resulted in a decrease of G6PD activity (Fig. 6i), which is consistent with the reported effect of PLK1 on activating G6PD activity^34^. Aligned with PLK1’s role in regulating GSIS, sh*PLK1* EndoC-βH1 cells exhibited reduced GSIS (Fig. 6j), as well as response to AZD7762 treatment (Fig. 6k). We further evaluated the role of G6PD in EndoC-βH1 cells by generating shRNA knockdown *G6PD*-deficient EndoC-βH1 cells (hereafter known as sh*G6PD* EndoC-βH1 cells; Supplementary Fig. 4c). Similar to the decrease in PLK1, the decrease in G6PD in EndoC-βH1 cells resulted in a reduction of GSIS (Fig. 6l) and GSIS potentiation by AZD7762 (Fig. 6m). In summary, these data demonstrate that reduced CHEK2 activity leads to a decrease in PP2A activity, resulting in an increase in PLK1 activity, followed by an elevation in G6PD activity (Extended Data Fig. 6e).

Interestingly, the effect of AZD7762 was glucose-dependent and CHEK2 phosphorylation was reduced in a glucose-dose-dependent manner (Supplementary Fig. 4d,e). Tolbutamide, a sulfonylurea drug that inhibits flux through the K_ATP_ channel and mimics glucose’s response, however, did not reduce CHEK2 phosphorylation (Supplementary Fig. 4f,g), confirming that inhibition of CHEK2 phosphorylation is not an event secondary to the closure of K_ATP_ channel and depolarization of the cell membrane. Consistent with other studies^35–39^, we observed an increase in G6PD activities and 6-phosphogluconate levels in MIN6 cells exposed to 20 mM glucose (Supplementary Fig. 4h,i). Bulk RNA sequencing (RNA-seq) on AZD7762-treated EndoC-βH1 cells showed that gene expression patterns between control and AZD7762-treated conditions are highly similar (Extended Data Fig. 6f). Furthermore, the volcano plot revealed that only a limited number of genes were significantly changed (Extended Data Fig. 6g). These evidence supporting the conclusion that AZD7762 primarily exerts its effects through the kinase pathway CHEK2–PP2A–PLK1–G6PD–PPP to acutely regulate GSIS response in β cells^40^. Nevertheless, we cannot exclude the possibility that other downstream targets of CHEK2 could be involved in regulating insulin secretion.

## Discussion

CHEK2 is classically known to be involved in DNA repair mechanisms. *CHEK2* variations have been associated with an increased risk of type 2 diabetes in multiple populations^41^. Checkpoint activation is also associated with low exocytosis in T2D β cells^42^. However, there are currently no reports of a direct link between CHEK2 and β-cell function. We identified CHEK2 inhibitors that effectively potentiated insulin secretion upon glucose stimulation using chemical screening. Improvements in glucose tolerance and in vivo insulin secretion were detected only in *CHEK2*^*−*/*−*^ mice that were fed with HFD. One possibility is that the loss of function of a protein from birth can be compensated developmentally^43–45^, which might mask the phenotype caused by the loss of gene function. Through untargeted metabolomics profiling, we discovered that inhibition of CHEK2 activity leads to activation of PPP that can potentially regulate GSIS^31,46^. Several studies have reported rapid increase in PPP metabolites in β cells after glucose exposure^1,30,36,37,47–50^. Moreover, extracellular glucose stimulates an increase in ribose-5-phosphate in β cells, indicating that β cells possess an active and responsive PPP^7,50,51^. Pathway perturbation study by removing function of the rate-limiting enzymes of PPP, G6PD, also resulted in impaired GSIS^50,52^. Furthermore, cholecystokinin-8, a gut hormone that potentiates GSIS, has been shown to increase PPP activity in pancreatic islets^53,54^; in clinical settings, G6PD deficiency is associated with an increase in the prevalence of T2D (ref. ^55^) and G6PD activities are reduced in T2D (ref. ^56^); patients with G6PD deficiency also have reduced insulin response to IVGTT^57^ and impaired fasting glucose^40,51,58,59^.

In summary, we discovered a previously unreported role of CHEK2 in modulating insulin secretion in both healthy and T2D conditions. While our study provides compelling evidence supporting the role of CHEK2 in AZD7762-potentiated insulin secretion in β cells, we recognize potential contribution of non-CHEK2 targets in this process, which will require further investigation. Due to the small chemical library used for screening in this study, only a limited number of new pathways were identified. Future research, using larger and diverse chemical libraries along with advanced screening techniques, holds promise for uncovering a broader array of therapeutic targets. Our findings strongly support the role of the PPP metabolic pathway in augmenting GSIS by inhibition of CHEK2. We used metabolomics and genetic manipulation strategies to discover a pathway that involves CHEK2, PP2A, PLK1 and G6PD in regulating insulin secretion. Manipulation of this axis could rescue the function of glucose-unresponsive β cells in T2D and enrich our comprehension of the molecular mechanism of GSIS regulation.

## Methods

All mouse studies have been approved by the Institutional Animal Care and Use Committee (IACUC) at Weill Cornell Medicine (2011-0024). All cynomolgus macaque work has been approved by IACUC at the University of Pennsylvania under protocol 806688. The pancreatic organs were obtained from the local organ procurement organization under the United Network for Organ Sharing (UNOS). The informed consent was obtained for research purposes.

### Cell lines

EndoC-βH1 cells (CVCL_L909; female) were purchased from EndoCells and cultured in DMEM containing 1 g l^−1^ glucose, 2% BSA fraction V (Sigma-Aldrich), 50 μM 2-mercaptoethanol, 10 mM nicotinamide (Calbiochem), 5.5 μg ml^−1^ transferrin (Sigma-Aldrich), 6.7 ng ml^−1^ selenite (Sigma-Aldrich), 100 U ml^−1^ penicillin and 100 μg ml^−1^ streptomycin. MIN6 cells (CVCL_0431) were provided by M. Hao (Weill Cornell Medicine) and cultured in DMEM containing 4.5 g l^−1^ glucose, 15% FBS (Gibco), 50 μM 2-mercaptoethanol, 2 mM glutamine (Gibco) and 100 U ml^−1^ penicillin and 100 μg ml^−1^ streptomycin. Cells were maintained at 37 °C with 5% CO_2_.

To generate NLuc-MIN6 and NLuc-EndoC-βH1 cells, we produced lentivirus expressing proinsulin-luciferase fusion protein from HEK293T cells (ATCC, CRL_3216), transfected with psPAX2 (Addgene, 12260), pMD2.G (Addgene, 12259) and proinsulin-NanoLuc (Addgene, 62057). We pooled viral supernatant collected at 48 h and 72 h post-transfection and concentrated the virus using Lenti-X Concentrator (Takara Bio). We added the concentrated virus to MIN6 or EndoC-βH1 cells in fresh culture medium with 8 μg ml^−1^ Polybrene (Sigma-Aldrich) and spun the cells at 800*g* for 1 h at 30 °C during infection. After 24 h, we placed cells in fresh growth media. Subsequently, we treated the infected EndoC-βH1 cells with 5 µg mL^−1^ blasticidin (Invitrogen) for 1 week to produce the stable NLuc-MIN6 and NLuc-EndoC-βH1 cell lines.

EndoC-βH1 *CHEK2*-deficient cells (sg*CHEK2* EndoC-βH1 cells) were generated by the lenti-CRISPR/Cas9 knockdown system. The sgRNA targets were obtained via the online program generated by Feng Zhang’s laboratory (http://crispr.mit.edu/). The sgRNA target sequences are listed in Supplementary Table 5. The lenti-CRISPR viruses were produced by transfecting the lenti-CRISPR/Cas9 plasmid (Addgene, 52961) along with psPAX2 and pMD2.G. For each 15-cm dish of 293T cells, 15 μg of lenti-CRISPR/Cas9, 9 μg of psPAX2 and 3 μg of the pMD2.G were transfected using polyethylenimine. Tissue culture media were refreshed 16 h after transfection, and media containing viruses were collected 48 h and 72 h after transfection. Viruses were then concentrated using the Lenti-X concentrator (Takara Bio). Infection protocol similar to that of NLuc-MIN6 and NLuc-EndoC-βH1 cells was used for generating EndoC-βH1 *CHEK2*-deficient cells. Cells infected by lentivirus were then cultured with 4 µg ml^−1^ puromycin (Invitrogen) for 1 week.

NLuc-EndoC-βH1 *CHEK2*-deficient cells (sg*CHEK2* NLuc-EndoC-βH1 cells) were generated by the lenti-CRISPR/Cas9 knockdown approach, as described above, by infecting NLuc-EndoC-βH1 cells with lentivirus carrying lenti-CRISPR/Cas9 plasmid with sequence either control scramble sgRNA or *CHEK2*-targeting sgRNA.

For knocking down AZD7762 target genes in EndoC-βH1 cells, we designed two different shRNAs to target each using the Broad Institute GPP Web Portal (https://portals.broadinstitute.org/gpp/public/). The shRNA target sequences are listed in Supplementary Table 8. Each sgRNA was then cloned into pLKO.1-blast (Addgene, 26655) and packaged along with psPAX2 and pMD2.G. The transfection and lentivirus infection protocol used was similar to that used for shRNA knockdown experiment, as described above. Cells infected by lentivirus were then cultured with 5 µg ml^−1^ blasticidin (Invitrogen) for 1 week.

Overexpression of *CHEK2* was achieved by infecting *CHEK2*-deficient sg*CHEK2* EndoC-βH1 with lentivirus carrying control or *CHEK2*-overexpression construct obtained from VectorBuilder.

### Bulk RNA-seq

Total RNA was extracted in TRIzol (Invitrogen) and treated with DNase I using the Directzol RNA Miniprep kit (Zymo Research). RNA-seq libraries of polyadenylated RNA were prepared using the TruSeq RNA Library Prep Kit v2 (Illumina) or TruSeq Stranded mRNA Library Prep Kit (Illumina). cDNA libraries were sequenced with pair-end 51 bps using an Illumina NovaSeq6000 platform. The resulting reads were checked for quality using FastQC (v0.10.1, https://www.bioinformatics.babraham.ac.uk/projects/fastqc) and were trimmed for adaptor sequences and low-quality bases using cutadapt (v1.18). To measure gene expression, the trimmed reads were aligned to the human reference genome (GRCh37). Raw gene counts were quantified using HTSeq-count (v0.11.2). The counts data were subjected to a regularized logarithm transformation using the rlog function within the DESeq2 package (v1.36.0). The transformed data were used to perform a principal component analysis (PCA) using the plotPCA function within the DESeq2 package. Additionally, the counts data were converted into fragment counts normalized per kilobase of feature-length per million mapped fragments (FPKM) using the fpkm function within the DESeq2 package, and an unsupervised hierarchical clustering on samples was conducted using the Pearson correlation coefficient metric. The R heatmap package (v1.0.12) was used to visualize the clustering result.

### Quantitative RT–PCR

To validate shRNA knockdown efficiency, we measured gene expression levels in control and knock-downed cells using quantitative RT–PCR. We isolated total RNA from EndoC-βH1 cells using the RNeasy Plus Universal Kit (Qiagen) and synthesized cDNA using the High-Capacity cDNA Reverse Transcription Kit (Applied Biosystems). We used a LightCycler 480 SYBR Green I Master System (Roche) in the quantitative RT–PCR experiments. We normalized the expression of target genes against Β actin. Primers used for quantitative RT–PCR experiments can be found in Supplementary Table 9.

### Insulin tolerance test

Before the insulin tolerance test, mice were fasted for 6 h. During the experiment, mice were injected IP with 1 IU kg^−1^ body weight of insulin and blood glucose levels were monitored at 0, 15, 30, 45, 60 and 120 min after insulin injection.

### Immunostaining

For immunostaining of human islets and mouse pancreatic section, tissue samples were fixed overnight in 4% paraformaldehyde (PFA) at 4 °C and incubated in primary antibody overnight at 4 °C. Samples were then washed with 1× phosphate-buffered saline (PBS) three times and then incubated in secondary antibodies and DAPI for 1 h at room temperature. Samples were then washed with 1× PBS three times. Immunostained tissue was then mounted in ProLong Gold Antifade Mountant (Molecular Probes). The primary antibodies and their respective concentration used in immunostaining experiments were as follows: goat anti-insulin antibody (Dako, Agilent, IR002; 1:50), sheep anti-NGN3 antibody (R&D Systems, AF3444; 1:200), rat anti-SST antibody (R&D Systems, MAB2358; 1:100), rabbit anti-glucagon antibody (Cell Signaling, 2760; 1:200), goat anti-polypeptide Y antibody (Novus Biologicals, NB-100-1793; 1:200) and rabbit anti-Ki67 antibody (Abcam, ab15580; 1:500). All secondary antibodies used are fluorescence-conjugated secondary antibodies (Alexa Fluor, Thermo Fisher Scientific). Microscopy images were obtained from Inverted Microscope/Apotome (Zeiss) and LSM 800 confocal microscope (Zeiss) and analyzed using ImageJ.

### Flow cytometry

EndoC-H1 or MIN6 cells were washed with PBS and resuspended in 300 μl PBS. In total, 5–10 μl of PI staining solution (Invitrogen, P3566), diluted to 10 μg ml^−1^, was added to control tubes and treated samples. Samples were mixed gently and incubated for 1 min in the dark. PI fluorescence was determined using an Attune Instrument in the YL-2 channel.

### PP2A activity

sg*CHEK2* and control EndoC-βH1 cells were starved in 0.5 mM glucose for 1 h and then stimulated with 20 mM glucose for 1 h. Cells were then lysed to assess PP2A activity with the PP2A Immunoprecipitation Phosphatase Assay Kit (MilliporeSigma).

### 6-Phosphogluconate levels

MIN6 cells were starved in 0 mM glucose for 1 h and then in 0 mM glucose for an additional 1 h. Then they were stimulated with either 0 mM or 20 mM glucose for 30 min. AZD7762 or control was included starting from the second starvation and throughout the rest of the experiments. Cells were lysed with Dounce homogenizer, and 6-phosphogluconate levels were assessed by 6-phosphogluconic acid Assay Kit (BioVision).

### H&E staining protocol

Slides were processed as follows for H&E staining: first, they were washed in PBS for 1 min and dipped in water once, then immersed in hematoxylin for 1 min and rinsed twice in water. After that, the slides were dipped in lithium carbonate once, washed again in water and then dipped in eosin. Next, the slides were dipped in 95% EtOH twice, followed by 100% EtOH twice, and then dipped two times in histoclear. Finally, the tissue sections on the slides were mounted using Cytoseal (Thermo Fisher Scientific).

### Focused chemical screen

A total of 2 × 10^5^ of MIN6 cells were cultured for 4 d and were starved in 0 mM glucose Krebs-Ringer Bicarbonate (KRBH) buffer for 1 h and then followed by an additional hour of starvation including the compounds. The source and purity of chemicals used in the screen are included in Supplementary Table 2. After the initial starvation, cells were treated with 20 mM glucose KRBH buffer for 30 min before 10 µl of supernatant was collected for assessing the luminescence levels using the Promega Nano-Glo Luciferase Assay System (N1120). The 96-well assay plate was then read by the Biotek Synergy H1 microplate reader.

### Pancreatic islets isolation

The islets were isolated in the Human Islet Core at the University of Pennsylvania following the guidelines of the Clinical Islet Transplantation Consortium protocol. Gift of Life as well as any other organ procurement organization who recovers the organs obtain consent from the deceased donor’s family. The collected organs could be used for research. There is no compensation for participants. All procedures are in compliance with the University of Pennsylvania IRB, Gift of Life leadership team and UNOS. Our research complies with all regulations and standards by the University of Pennsylvania Institutional Review Board, which is responsible for approval of the protocol. Cynomolgus macaque islet isolation was performed based on a modified protocol using Liberase enzyme (Roche). The islets were purified from the digested pancreas using a three-layer, discontinuous Euro-Ficoll gradient (densities 1.108, 1.096 and 1.037) and a COBE blood cell processor (COBE Laboratories). Samples were collected from different layers after islet purification to assess the purity of cell isolation. Final samples were stained with dithizone, counted manually and sized using a formula to calculate islet number and islet equivalents based on a 150-mm diameter. Islet preparations with purity >85% were used for this study. The isolated islets were cultured overnight in CRML 1066 (Mediatech) containing 10% heat-inactivated FBS at 25–28 °C in 95% O_2_ and 5% CO_2_. Mouse islets were isolated following a previously published protocol^60^.

### Mouse models

C57BL/6J (stock 000664), B6.Cg-*Lep*^*ob*^/J (commonly referred to as *ob*/*ob*, stock 000632), mice were obtained from the Jackson Laboratory. C57BL/6 N-A^*tm1Brd*^*CHEK2*^*tm1b(EUCOMM)Hmgu*/^JMmucd (stock: 047090-UCD, also known as *Chk2*) mice were acquired from the Knockout Mouse Project repository at the Jackson Laboratory. CD-1/ICR mice (stock 022) were obtained from the Charles River Laboratories. For HFD studies, 8-week-old C57BL/6J mice were fed with 60% HFD (Research Diets, D12492i) for 4 months. For *Chk2*^*−*/*−*^ mice HFD studies, 8-week-old wild-type and *Chk2*^*−*/*−*^ mice were fed with 60% HFD (Research Diets, D12492i) for 6 months. Unless stated otherwise, all mice were maintained on a normal chow diet and a 12-h light/12-h dark cycle in a pathogen-free animal facility, where ambient temperature was consistently maintained at 25 °C, with humidity levels ranging between 30% and 70%.

For in vivo studies with AZD7762, AZD7762 is dissolved in 11.3% (2-hydroxypropyl)-β-cyclodextrin. All animals were fasted overnight and treated with AZD7762 IP 1 h before the experiments. For mouse GTT and GSIS, 12 or 25 mg kg^−1^ AZD7762 was given by IP injection, and then 0.5–2 g kg^−1^ glucose was given either by IP injection or orally. Glucose levels were measured at −60, 0, 15, 30, 60, 90 and 120 min postglucose. For GSIS experiments, blood was drawn from the tail vein at −60, 0, 15 and 30 min postglucose. For HOMA-IR calculation for vehicle- and AZD7762-treated CD-1/ICR, HFD-fed C57BL/6J mice and *ob*/*ob* mice, we used the method as described in ref. ^61^.

### Cynomolgus macaque models

Cynomolgus macaque experiments are approved under the animal Protocol title—Cellular approaches for the modulation of alloresponses in nonhuman primates (protocol 806688). Adult Mauritius-origin male cynomolgus macaques (*Macaca fascicularis*) were provided by Alpha Genesis. After overnight fasting, the animal was sedated with ketamine (3–4 mg kg^−1^) mixed with dexmedetomidine (0.15 mg kg^−1^ intramuscular). Baseline blood samples were collected before the IV infusion of vehicle or 1.55 mg kg^−1^ AZD. Blood glucose was monitored at −20 and −40 min after infusion. At time 0 min, glucose (0.5 g kg^−1^ body weight) was infused IV via the IV catheter. Blood glucose was analyzed using a bedside glucometer (whole blood), and serum was tested for insulin/C-peptide levels. A small blood drop (~0.3 µl) was produced by a pinprick (using either a lancet or a needle) at 1, 3, 5, 7, 10, 15, 20, 30 and 60 min after administration of glucose. Additional blood samples (0.5 ml) were collected at 0, 1, 3, 5, 10, 15 and 30 min to measure insulin/C-peptide levels.

### Insulin secretion assays

For human, mouse and cynomolgus macaque islets experiment, islets were starved in 2 mM glucose KRBH buffer for 2 h at 37 °C, and then stimulated in 2 mM glucose KRBH buffer for 1 h and subsequently with 20 mM glucose KRBH buffer for 1 h. To measure the total level of insulin in samples, cells were lysed in 0.1% Triton X-100. MIN6 cells were starved in 0 mM glucose for 1 h and then in 0 mM glucose for an additional 1 h. Then the cells were stimulated with either 0 mM or 20 mM glucose for 30 min. EndoC-βH1 cells were starved in 0.5 mM glucose for 1 h and then stimulated with either 0.5 mM or 20 mM glucose for 1 h. AZD7762 was included during the starvation and glucose stimulation steps. For MIN6 experiments, AZD7762 was included starting from the second starvation and throughout the rest of the experiments. For pseudoislet experiments, 4,000 EndoC-βH1 cells or 2,000 dissociated human islet cells were aggregated in v-bottom plate in culture media for 4 d before GSIS. A total of 50 mM KCl was used for KCl-stimulated insulin secretion experiments. At the end of each stimulation, 100 µl buffer was collected to assess insulin or C-peptide levels with ELISA kits (Alpco and Novus Biologicals). The islet perifusion experiment was carried out in a BioRep Perifusion System. During perifusion dynamic GSIS experiments, islets or pseudoislets were perfused at 100 µl min^−1^ of KRBH buffer containing 2 mM glucose for 30 min, then 45 min with 20 mM glucose and 15 min with 2 mM glucose again, and finally with 50 mM KCl KRBH buffer for 15 min. Samples were collected for assessment of insulin and C-peptide levels every 90 s. Data were normalized to baseline insulin secretion at 2 mM glucose for human islets and 0.5 mM for EndoC-βH1 cells.

### Hormones measurement

Proinsulin levels were measured using Total Proinsulin Chemiluminescence ELISA Kit (Alpco). Human insulin levels were measured by Insulin Chemiluminescence ELISA Kit (Alpco). Human C-peptide levels were measured by C-Peptide Chemiluminescence ELISA Kit (Alpco). Mouse insulin levels were measured by Rodent Insulin Chemiluminescence ELISA Kit (Alpco). Glucagon levels were measured by Glucagon ELISA (Alpco). Total GLP-1 ELISA (7–36 and 9–36; Alpco) and active GLP-1 levels were measured by Active GLP-1 (7–36) Amide Chemiluminescence ELISA (Alpco). Total GIP levels were measured by Total GIP ELISA (Alpco). SST levels were measured by SST (Human, Rat, Mouse, Porcine)—ELISA Kit (Phoenix Pharmaceuticals).

### Western blot

Protein was extracted from EndoC-βH1 cells in radioimmunoprecipitation assay buffer (Sigma-Aldrich) supplemented with halt protease and phosphatase inhibitor Cocktail (Thermo Fisher Scientific). Protein samples were loaded onto NuPAGE 4–12% Bis–Tris Protein Gels (Thermo Fisher Scientific), resolved by electrophoresis and transferred onto nitrocellulose membranes. Membranes were incubated with the following primary antibodies: mouse monoclonal anti-β-actin antibody (Invitrogen, MA1-140; 1:20,000), rabbit monoclonal anti-phospho CHEK2 T68 antibody (Cell Signaling, 2197S; 1:1,000), mouse monoclonal anti-CHEK2 antibody (Cell Signaling, 3440T; 1:1,000), rabbit anti-eIF2α antibody (Cell Signaling, 5324; 1:1,000), rabbit anti-phospho eIF2α Ser51 antibody (Cell Signaling, 3597; 1:1,000), rabbit anti-α-tubulin antibody (Cell Signaling, 2144; 1:1,000), rabbit anti-phospho PLK1 T210 (Abcam, ab155095; 1:200) and mouse anti-PLK1 (Abcam, ab17056; 1:1,000). Primary antibodies were detected by fluorophore-conjugated secondary goat anti-rabbit (LI-COR IRDye 800CW, 926-32213; 1:15,000) and donkey anti-mouse (LI-COR IRDye 680RD, 926-32210; 1:15,000) using LI-COR Odyssey Imagers. Western blot images were analyzed with Image Studio software 5.2.5.

### Cytosolic NADPH/NADP ratio and G6PD activities

Twenty million MIN6 cells were cultured for 4 d and lysed in 300 µl homogenization buffer. Cytosolic fraction was separated from the mitochondria fraction with a mitochondria extraction kit (Novus Biologicals) and used directly for measuring NADPH/NADP levels (Promega). For experiments measuring G6PD activities, 2 million MIN6 cells were cultured for 4 d and lysed in 300 µl PBS. G6PD levels were measured with a G6PD Activity Assay Kit (Abcam, Fluorometric).

### Untargeted metabolomics

MIN6 cells were starved in 0 mM glucose for 1 h and then in 0 mM glucose for an additional 1 h. Then they were stimulated with either 0 mM or 20 mM glucose for 30 min in the presence of 0 or 10 µM of AZD7762 throughout the experiment. The samples were homogenized with 300 µl of methanol:water (4:1, vol/vol), containing three internal standards, 1 nM U13_succinate, 1 nM U13_citrate and 0.5 nM heptadecanoic acid. Then, the samples were quickly frozen on liquid nitrogen and thawed on ice. The thawed samples were sonicated for 2 min. The freeze-thaw-sonication procedure was repeated three times. After that, the samples were kept at −20 °C for 10 min and then centrifuged at 13,500*g* for 10 min. Then, 250 µl of supernatant was transferred to a sampling vial. The reminder supernatant from each sample was put together to make the pooled quality control (QC) sample. The samples were dried under gentle nitrogen flow and derivatized with a two-step derivatization procedure. First, the samples were methoximized with 50 µl of methoxyamine hydrochloride (15 mg ml^−1^ in pyridine) at 30 °C for 90 min. The silylation step was done with 50 µl of N,O-bis(trimethylsilyl)trifluoroacetamide (containing 1% trimethylchlorosilane) at 70 °C for 60 min. QC sample was run multiple times during the analysis. The samples were analyzed by gas chromatography time-of-flight mass spectrometry (GC-TOFMS Premier, Waters). A number of 491 variables were detected after alignment and excluded any known artificial peaks. The data were normalized to the intensity of the sum of all the metabolites. The dataset was then imported into SIMCA-p software for PCA and PLS-DA. Variable importance in the projection (VIP) values from PLS-DA model were used for metabolites selection (VIP > 1). Heatmap was generated with R Studios 2021.09.0 with R 4.1.2 and pheatmap 1.0.12.

### Kinase profiling

The kinase profiling was performed using the SelectScreen Biochemical Kinase Profiling Service by Thermo Fisher Scientific at a concentration of 1 µM AZD7762.

### Statistics and reproducibility

No statistical methods and power analysis were used to predetermine sample sizes, but our sample sizes are similar to those reported previously^6^. For in vitro studies, *n* = 3 independent replicates or 3 individual participants or donors were used for all experiments unless stated otherwise. All experiments were independently repeated at least three times with similar results. Data are shown as mean ± s.e.m. For two-group data, we used a two-tailed unpaired Student’s *t*-test. For one independent variable data, we used one-way analysis of variance (ANOVA, Dunnett’s test). For experiments in Fig. 5 that involved two control and two experimental groups, we used mixed ANOVA for statistical analysis. Statistical analysis was performed using GraphPad Prism 9 software. Data distribution was assumed to be normal, but this was not formally tested. Details for mouse islets immunostaining analysis are included in Supplementary Table 10.

### Reporting summary

Further information on research design is available in the Nature Portfolio Reporting Summary linked to this article.

## Online content

Any methods, additional references, Nature Portfolio reporting summaries, source data, extended data, supplementary information, acknowledgements, peer review information; details of author contributions and competing interests; and statements of data and code availability are available at 10.1038/s41589-023-01466-4.

### Supplementary information

Supplementary Information Supplementary Figs. 1–7 and Supplementary Tables 1–10.

Reporting Summary

### Source data

Source Data Fig. 1 Statistical source data.

Source Data Fig. 2 Statistical source data.

Source Data Fig. 3 Statistical source data.

Source Data Fig. 4 Statistical source data.

Source Data Fig. 4 Unmodified blots.

Source Data Fig. 5 Statistical source data.

Source Data Fig. 6 Statistical source data.

Source Data Fig. 6 Unmodified blots.

Source Data Extended Data Fig. 1 Statistical source data.

Source Data Extended Data Fig. 2 Statistical source data.

Source Data Extended Data Fig. 3 Statistical source data.

Source Data Extended Data Fig. 4 Statistical source data.

Source Data Extended Data Fig. 5 Statistical source data.

Source Data Extended Data Fig. 6 Statistical source data.

## Data Availability

All data needed to evaluate the conclusions in the paper are present in the paper and/or the Supplementary Information. All raw data are available through source data files and have been uploaded to Mendeley (10.17632/n4bssm8kww.1). The raw RNA-seq data generated in this study have been deposited in the Gene Expression Omnibus database under accession GSE239335. Source data are provided with this paper.
