# Supplementary material for: Checkpoint kinase 2 controls insulin secretion and glucose homeostasis
Source: Nat Chem Biol. 2023 Nov 9;20(5):566–76. doi: 10.1038/s41589-023-01466-4 (PMC11062908; doi:10.1038/s41589-023-01466-4)
Supplement: Supplementary file 1 — Supplementary Figs. 1–7 and Supplementary Tables 1–10. [file 41589_2023_1466_MOESM1_ESM.pdf]

nature chemical biology

Article https://doi.org/10.1038/s41589-023-01466-4

# Checkpoint kinase 2 controls insulin secretion and glucose homeostasis

In the format provided by the authors and unedited

# Supplementary Figure 1

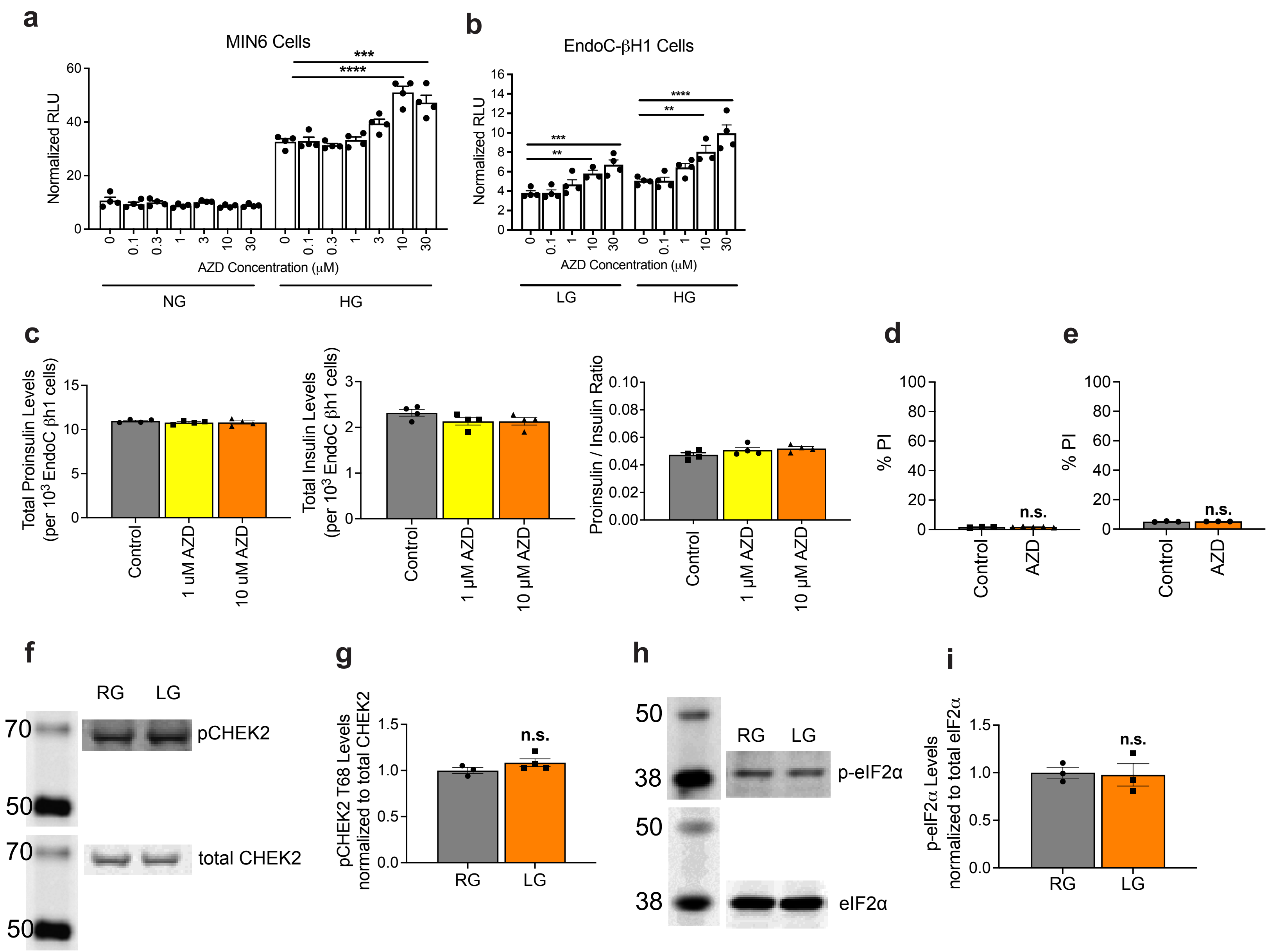

**Supplementary Fig. 1. AZD7762 increases insulin secretion of β cells.** (**a**) Nano-luciferase (NLuc) secretion during GSIS of NLuc-MIN6 cells in the presence of 0, 0.1, 0.3, 1, 3, 10, or 30 μM of AZD7762. NG: 0 mM glucose; HG: 20 mM glucose. n = 4 biological replicates. *P*-value: 30 μM of AZD7762, p = 2.50E-05; 10 μM of AZD7762, p = 3.52E-07. (**b**) NLuc secretion of NLuc-EndoC-βH1 cells during GSIS in the presence of 0, 0.1, 1, 10, or 30 μM of AZD7762. LG: 0.5 mM glucose; HG: 20 mM glucose. *P*-value: LG 10 μM of AZD7762, p = 0.0158; LG 30 μM of AZD7762, p = 0.0004; HG 10 μM of AZD7762, p = 9.67E-03; HG 30 μM of AZD7762, p = 8.96E-05. n = 4 biological replicates. (**c**) Intracellular proinsulin levels, intracellular insulin levels, and proinsulin to insulin ratio of EndoC-βH1 cells in the presence of 0, 1 or 10 μM AZD7762. n = 4 biological replicates. (**d**) The percent of PI-stained EndoC-βH1 cells after 1 hour treatment with control or 1 μM AZD7762. n = 3 (Control) and n = 5 (AZD7762) biological replicates. (**e**) The percent of PI-stained EndoC-βH1 cells after 24-hour treatment with control or 1 μM AZD7762. n = 3 (Control) and n = 3 (AZD7762) biological replicates. (**f** and **g**) Western blotting (f) and the quantification (g) of phospho-CHEK2 Thr68 of EndoC-βH1 cells in 5 mM glucose (RG) and 0.5 mM glucose (LG). n = 3 (RG) and n = 4 (LG) biological replicates. (**h** and **i**) Western blotting (h) and the quantification (i) of phospho-eIF2α of EndoC-βH1 cells in 5 mM glucose (RG) and 0.5 mM glucose (LG). n = 3 biological replicates. For a-c, *P*-value was calculated by one-way ANOVA. For d-e, g and i, *P*-value was calculated by two-sided Student's *t*-test. Data represent the Mean ± SEM. Statistical significance $p < 0.001$, ***; $p < 0.0001$, ****; n.s. is not significantly different.

# Supplementary Figure 2

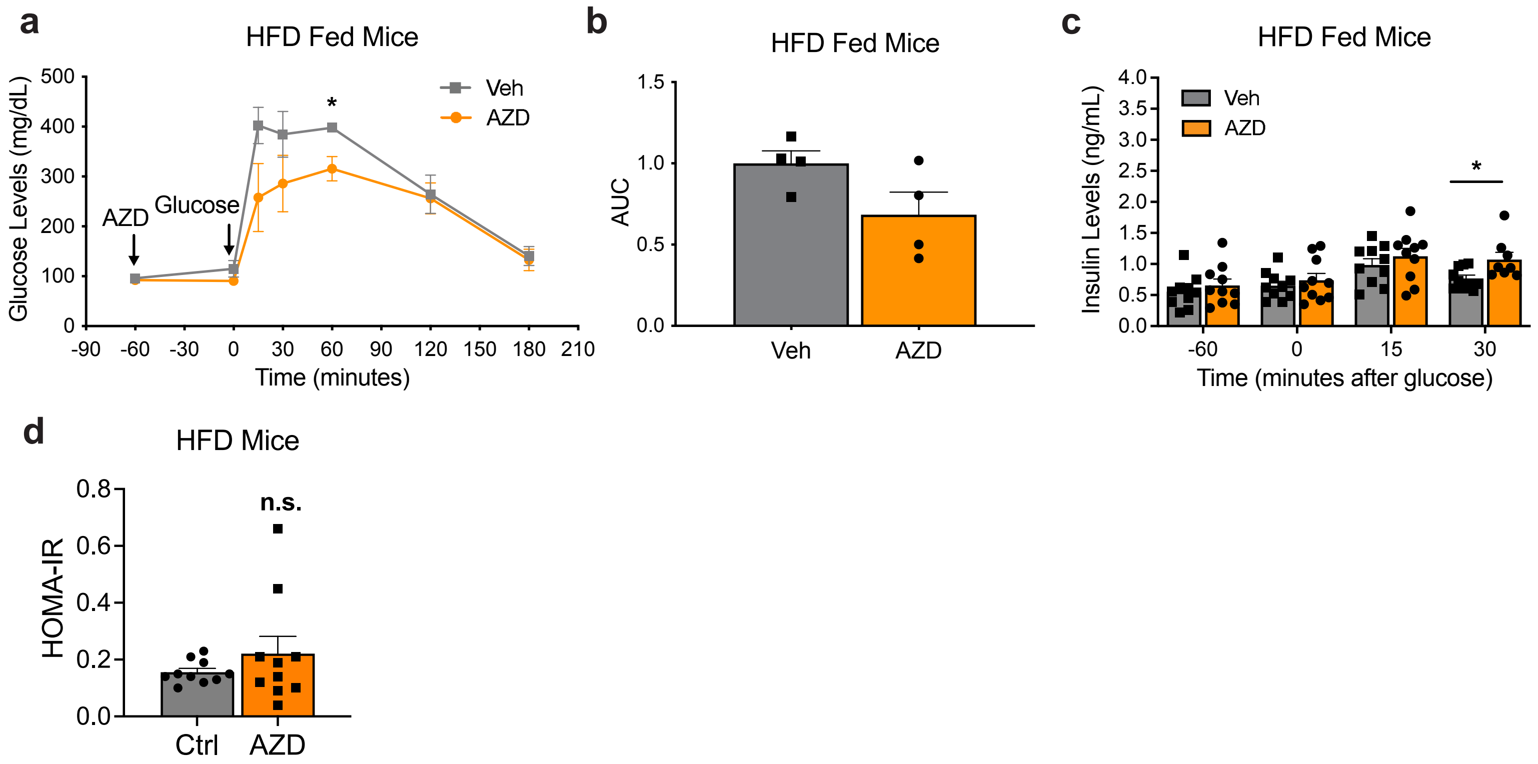

**Supplementary Fig. 2. AZD7762 improves glucose tolerance and increases insulin secretion in HFD C57Bl6/J mice.** (**a** and **b**) IPGTT (a) and AUC (b) of 6-month-old 60% HFD C57Bl6/J mice treated with vehicle or 25 mg/kg AZD7762. n = 4 vehicle-treated mice; n = 4 AZD7762-treated mice. (**c**) GSIS of 6-month-old 60% HFD C57BL6/J mice treated with vehicle or 25 mg/kg AZD7762. n = 10 vehicle-treated mice; n = 8 AZD7762-treated mice. *P*-value = 0.038. (**d**) HOMA-IR of 6-month-old 60% HFD C57Bl6/J mice treated with vehicle or 25 mg/kg AZD7762. n = 10 mice per group. *P*-value was calculated by two-sided Student's *t*-test unless otherwise stated. Data represent the Mean ± SEM. Statistical significance $p < 0.05$, *.

# Supplementary Figure 3

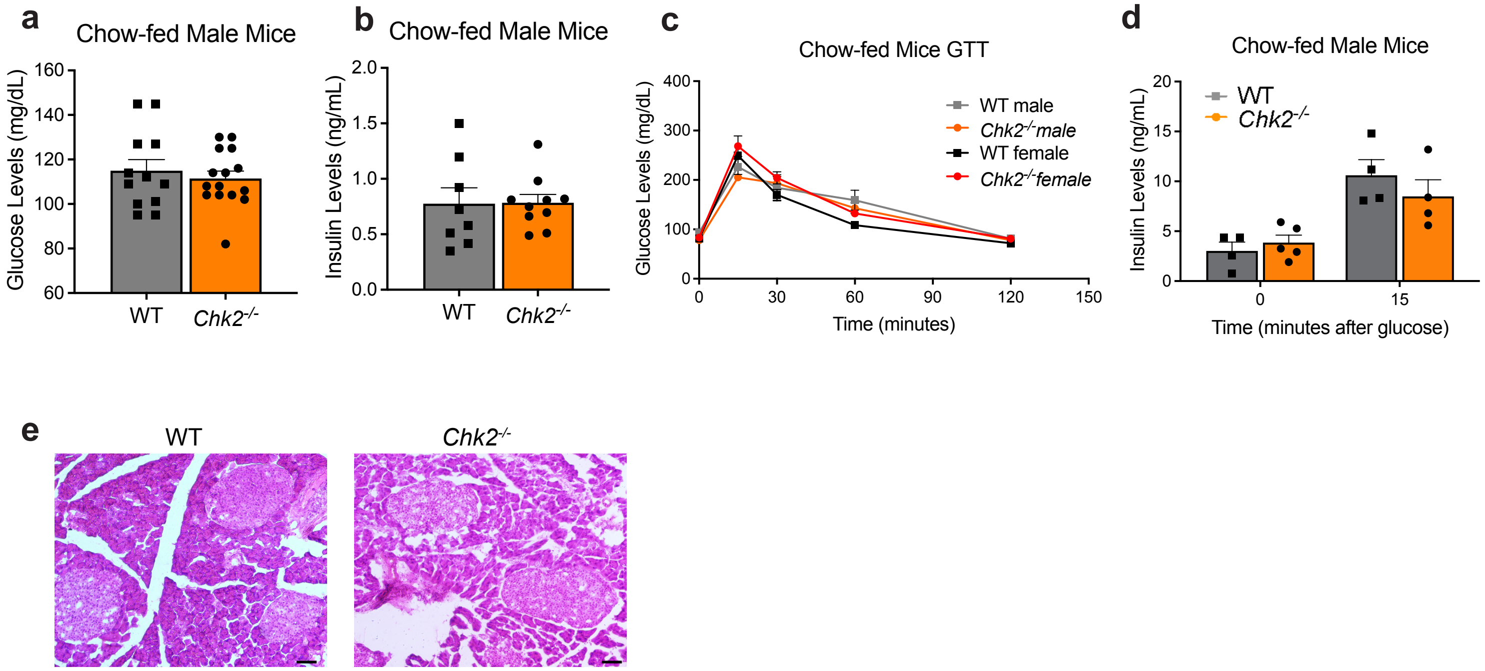

**Supplementary Fig. 3. Chow-fed *Chk2*$^{-/-}$ mice show unchanged GSIS and glucose homeostasis.** (**a**) Fed glucose levels in male 8-month-old chow-fed wildtype and *Chk2*$^{-/-}$ mice. n ≥ 12 mice per group. n = 12 wildtype mice; n = 15 *Chk2*$^{-/-}$ mice. (**b**) Fed insulin levels of adult 8-month-old chow-fed wildtype and *Chk2*$^{-/-}$ mice. n = 8 wildtype mice; n = 10 *Chk2*$^{-/-}$ mice. (**c**) Glucose levels during oral glucose tolerance test (OGTT) of 8-month-old chow-fed wildtype and *Chk2*$^{-/-}$ mice. n = 7 wildtype female mice; n = 6 *Chk2*$^{-/-}$ female mice; n = 4 wildtype male mice; n = 5 *Chk2*$^{-/-}$ male mice. (**d**) Insulin levels during OGTT of 8-month-old male chow-fed wildtype and *Chk2*$^{-/-}$ mice. n = 4 wildtype mice; n = 4 *Chk2*$^{-/-}$ mice. (**e**) H&E staining of pancreatic section of 8-month-old male HFD-fed wildtype and *Chk2*$^{-/-}$ mice. n = 3 independent experiments. Scale Bar: 50 µm. *P*-value was calculated by two-sided Student's *t*-test unless otherwise stated. Data represents the Mean ± SEM.

# Supplementary Figure 4

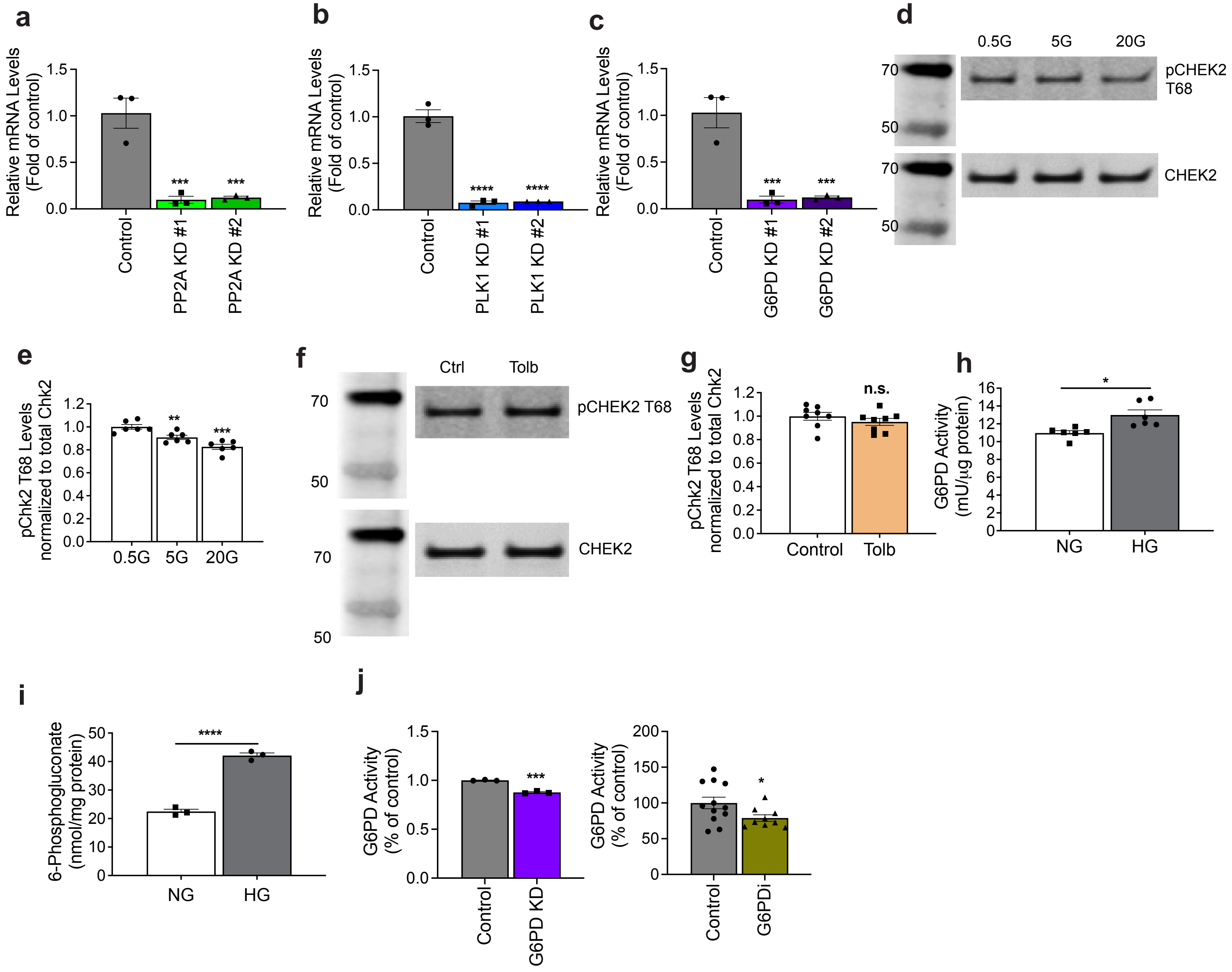

**Supplementary Fig. 4. CHEK2/pentose phosphate axis is involved in insulin secretion.** (**a**) Quantitative RT-PCR for *PP2A* in control and sh*PP2A* EndoC-βH1 cells. P-value: Control vs PP2A KD #1, p = 0.024; Control vs PP2A KD #2, p = 0.029. n = 3 biological replicates. Gene expression was normalized to that of β-Actin and presented as fold changes (± SEM) against control expression. (**b**) Quantitative RT-PCR for *PLK1* in control and sh*PLK1* EndoC-βH1 cells. n = 3 biological replicates. Gene expression was normalized to that of β-Actin and presented as fold changes (± SEM) against control expression. (**c**) Quantitative RT-PCR for *G6PD* in control and sh*G6PD* EndoC-βH1 cells. n = 3 biological replicates. Gene expression was normalized to that of β-Actin and presented as fold changes (± SEM) against control expression. (**d** and **e**) Western blotting (d) and the quantification (e) of phospho-CHEK2 Thr68 in EndoC-βH1 cells treated with 0.5 mM, 5 mM or 20 mM glucose. n = 6 biological replicates. (**f**) and (**g**) Western blotting (f) and the quantification (g) of phospho-CHEK2 Thr68 in EndoC-βH1 cells treated with 500 μM tolbutamide. n = 8 biological replicates. (**h**) G6PD activity in MIN6 cells stimulated with 0 mM (NG) or 20 mM glucose (HG). n = 6 biological replicates. (**i**) A pentose phosphate pathway intermediate, 6-phosphogluconate levels in MIN6 cells stimulated with 0 mM glucose (NG) or 20 mM glucose (HG). n = 3 biological replicates. (**j**) G6PD activity. Left: Control and sh*G6PD* EndoC-βH1 cells. n = 3 biological replicates. *P*-value was calculated by two-sided Student's *t*-test unless otherwise stated. For a-c and e, *P*-value was calculated by one-way ANOVA. Data represents the Mean ± SEM. Statistical significance $p < 0.05$, *; $p < 0.001$, ***, $p < 0.0001$, ****.

# Supplementary Figure 5

**a**

**Raw Data for Supplementary Figure 1f**

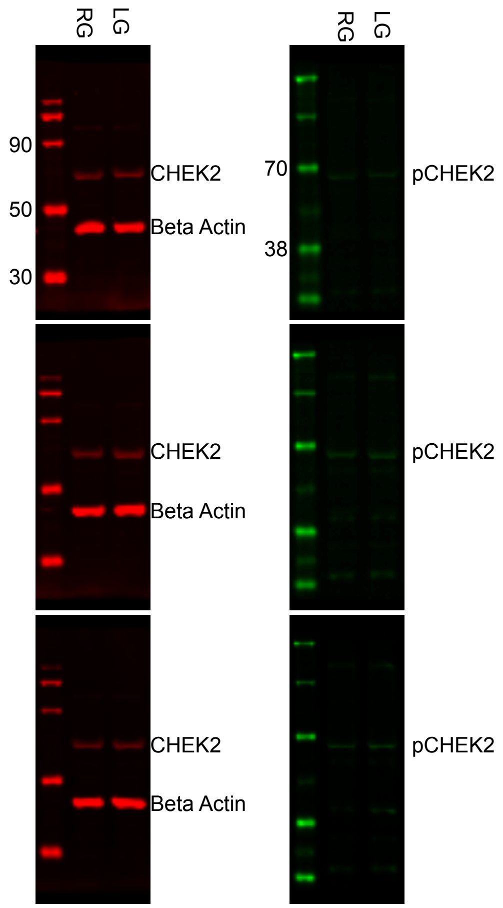


**b**

**Raw Data for Supplementary Figure 1h**

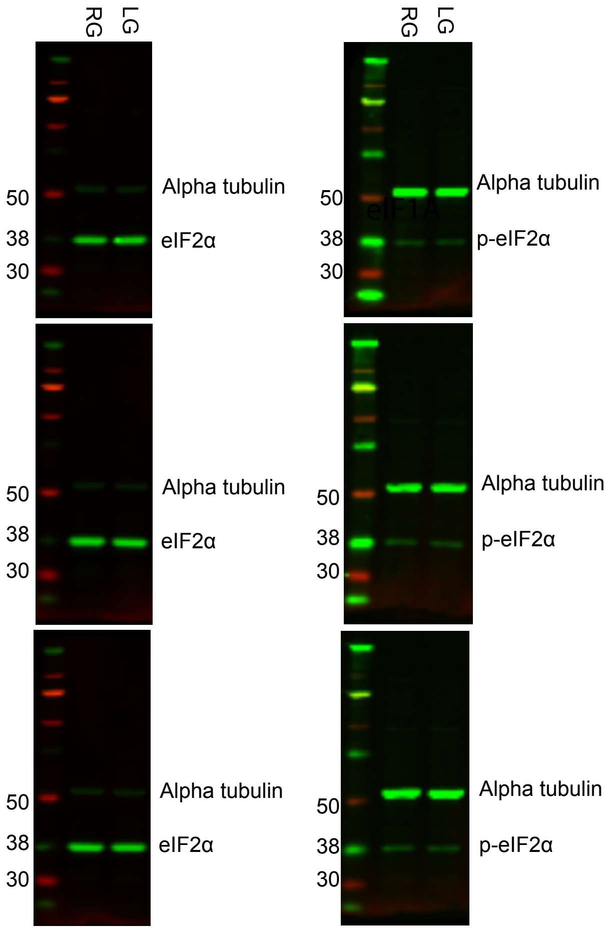

**Supplementary Fig. 5. Raw data for Western blots in Supplementary Figure 1.** Uncropped images of western blots displayed in Supplementary Fig. 1f (**a**) and Supplementary Fig. 1h (**b**).

# Supplementary Figure 6

**a**

**Raw Data for Supplementary Figure 4d**

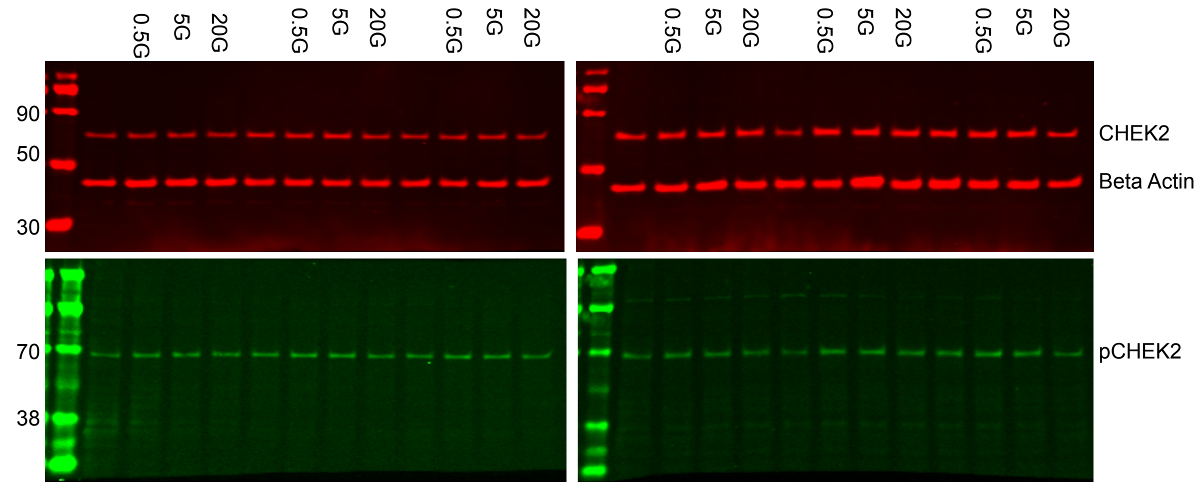


**b**

**Figure 4f**

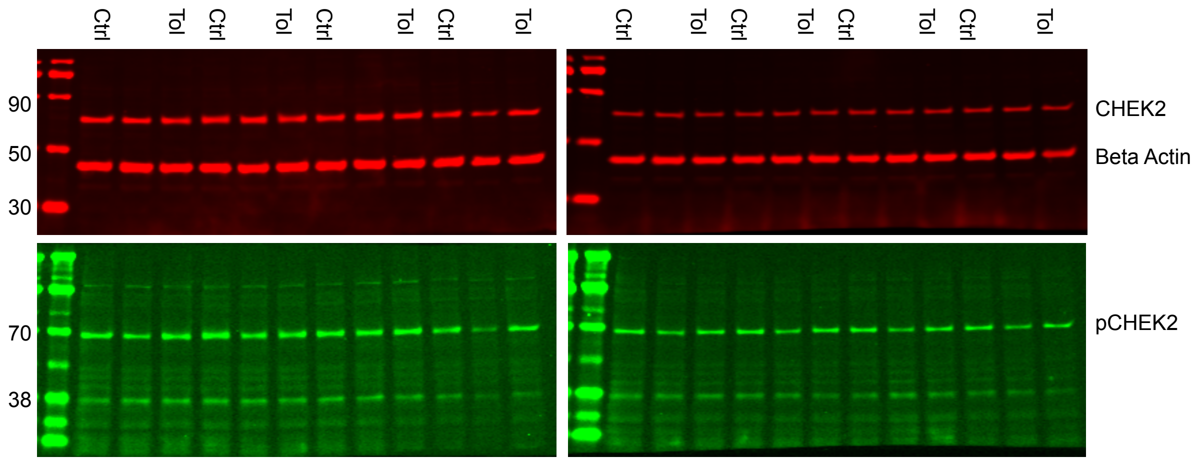

**Supplementary Fig. 6. Raw data for Western blots in Supplementary Figure 4.** Uncropped images of western blots displayed in Supplementary Fig. 4d (**a**) and Supplementary Fig. 4f (**b**).

# Supplementary Figure 7

**a**

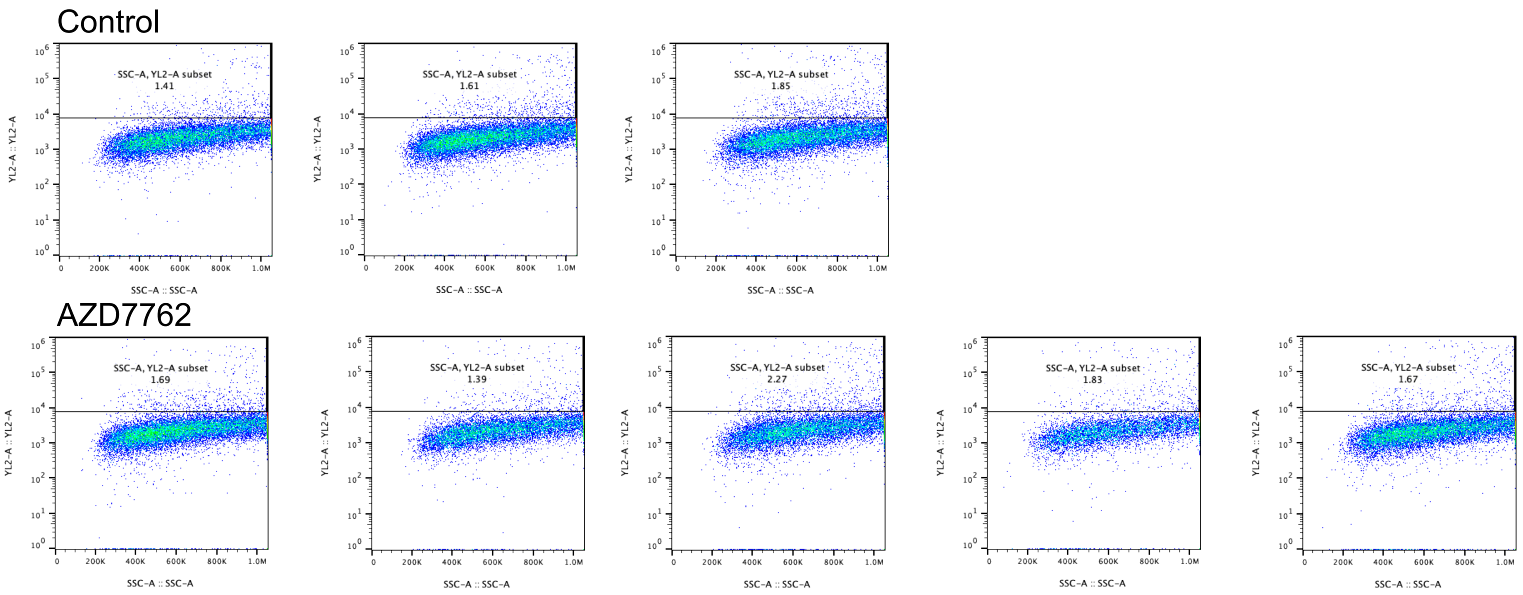


**b**

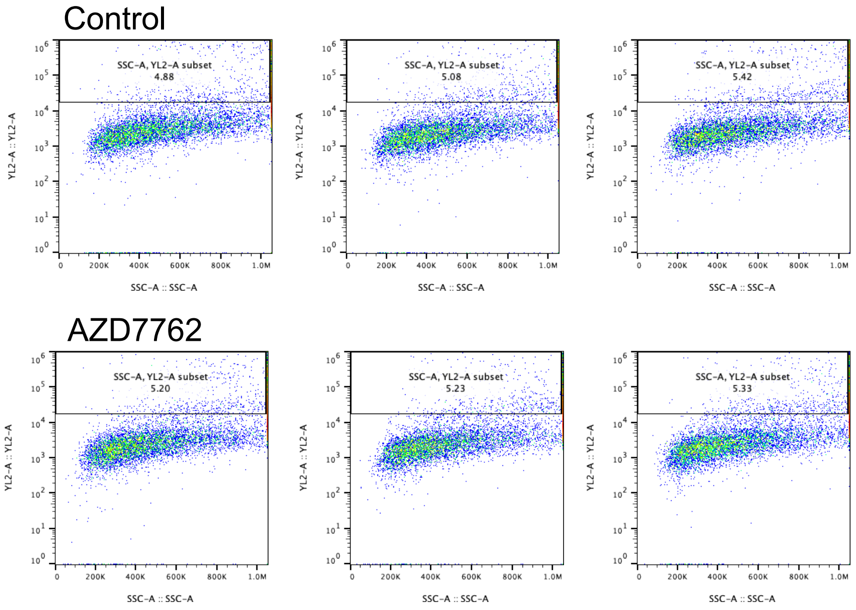

**Supplementary Fig. 7. Raw data for Flow Cytometry in Supplementary Figure 1.** Flow cytometry images for SI Fig 1d (**a**) and SI 1e (**b**).

**Supplementary Table 1. Primary screening data.**

| **Category** | **Parameter** | **Description** |
|---|---|---|
| Assay | Type of assay | Cell-based luciferase reporter; NLuc-MIN6 cells |
| | Target | Insulin secretion upon stimulation with 20 mM glucose |
| | Primary measurement | Luminescence signals |
| | Key reagents | Promega Nano-Glo Luciferase Assay System (Cat # N1120) |
| | Assay protocol | See **Methods** |
| | Additional comments | |
| Library | Library size | 223 compounds |
| | Library composition | In-house chemical library |
| | Source | Commercial sources |
| | Additional comments | |
| Screen | Format | 96 well |
| | Concentration(s) tested | 10 μM |
| | Plate controls | Vehicle (DMSO) |
| | Reagent/ compound dispensing system | Manual pipetting |
| | Detection instrument and software | Biotek Synergy H1, Gen5 |
| | Assay validation/QC | Repeat assays and secondary assays |
| | Correction factors | None applied |
| | Normalization | |
| | Additional comments | |
| Post-HTS analysis | Hit criteria | > 1.5 fold |
| | Hit rate | 9.4 % |
| | Additional assay(s), | validate with insulin secretion |
| | Confirmation of hit purity and structure | Confirmation of activity in quadruplicates in human NLuc-EndoC-βH1 cells, increased in insulin concentration was directly measured by ELISA |
| | Additional comments | |

**Supplementary Table 2. Compounds in the focused chemical library.**

| Small molecules | Vendor | Catalog No. | Purity (%) |
|---|---|---|---|
| (+)-Aeroplysinin-1 | MedChemExpress | HY-19827 | ≥95.0% |
| 2-Methoxyestradiol | Millipore Sigma | M6383 | ≥ 98.0% (TLC) ≥ 98.0% (HPLC) |
| 6-bromoindirubin-3'-acetoxime(BIO-Acetoxime) | MedChemExpress | HY-15356 | ≥98.0% |
| 6-bromoindirubin-3'-oxime (BIO) | MedChemExpress | HY-10580 | 99.74% |
| A 83-01 ((TGF-β RI Inhibitor IV) | Tocris | 2939 | ≥98% (HPLC) |
| AB 1010 (Masitinib mesylate) | MedChemExpress | HY-10209A | 99.83% |
| AGL 2043 | Santa Cruz Biotechnology | 22617-28-8 | NA |
| Akt Inhibitor VIII, Isozyme-Selective, Akti-1/2 | Millipore Sigma | 124018 | ≥95% by HPLC |
| ALK5 Inhibitor I TbR-I Inhibitor Transforming Growth Factor-b Type I Receptor Kinase Inhibitor (LY 364947) | MedChemExpress | SB-431542 | 99.89% |
| Alsterpaullone | Tocris | 6400 | ≥98% (HPLC) |
| AMPK Inhibitor, Compound C, Dorsomorphin | Millipore Sigma | P5499 | ≥98% (HPLC) |
| Anadamide (AEA) | Avanti Polar Lipids | 870430 | >99% |
| AR-A0144-18 | Tocris | 3966 | ≥99% (HPLC) |
| Aurora Kinase Inhibitor II (N-[4-[(6,7-Dimethoxy-4-quinazolinyl)amino]phenyl]benzamide hydrochloride) | Millipore Sigma | D6068 | ≥98% (HPLC), solid |
| AY 9944 | MedChemExpress | HY-107420 | 99.71% |
| AZD7762 | Selleckchem | S1532 | 99.98% |
| Bay 11-7821(Bay 11-7082) | Selleckchem | S2913 | 99.98% |
| Bay11-7085 | Selleckchem | S7352 | 99.87% |
| BCI | Selleckchem | S2837 | 99.88% |
| Betulinic acid | Millipore Sigma | B8936 | ≥98% (HPLC) |
| BIBF1120 | MedChemExpress | HY-50904 | 99.94% |
| BIBU 1361 | Tocris | 2417 | ≥97% (HPLC) |
| BIBW 2992 | Selleckchem | S1011 | 99.95% |
| BIBX 1382 | MedChemExpress | HY-10322 | 99.05% |
| BML-277 | Selleckchem | S8632 | 99.17% |

| | | | |
|---|---|---|---|
| BMP Inhibitor LDN-193189 (DM-3189) | MedChemExpress | HY-12071 | 99.42% |
| BPDQ | Santa Cruz Biotechnology | 169205-87-2 | NA |
| BPIQ-I | Cayman Chemicals | 174709-30-9 | ≥95% |
| Brivanib | Selleckchem | S1084 | 99.54% |
| Butein | Selleckchem | S8036 | 99.98% |
| Caffeic acid phenethyl ester (CAPE) | Millipore Sigma | C8221 | ≥97% (HPLC) |
| Carnosol | Selleckchem | S9127 | 99.75% |
| Casein Kinase I Inhibitor, D4476 | MedChemExpress | HY-10324 | 99.94% |
| CAY10470 (QNZ) | Cayman Chemicals | 545380-34-5 | ≥98% |
| CCT 241533 | Tocris | 4968 | ≥98% (HPLC) |
| Celastrol | Millipore Sigma | C0869 | ≥98% (HPLC) |
| CHIR-258 Dovitinib | Selleckchem | S1018 | 99.98% |
| CHIR99021 | Tocris | 4423 | ≥98% (HPLC) |
| CI 1033 - Canertinib dihydrochloride | Tocris | 5916 | ≥98% (HPLC) |
| Cilostamide | Millipore Sigma | C7971 | ≥97% (HPLC) |
| Compound 56 | Santa Cruz Biotechnology | 171745-13-4 | ≥97% |
| CP-690550 Tofacitinib | Selleckchem | S5001 | 99.99% |
| Cryptotanshinone | Millipore Sigma | C5624 | ≥98% (HPLC) |
| Cucurbitacin I | Cayman Chemicals | 2222-07-3 | ≥98% |
| Curcumin | Millipore Sigma | C1386 | ≥65% (HPLC) |
| Cycloheximide | Millipore Sigma | 01810 | ≥90% (HPLC) |
| Cyclopamine-KAAD (Shh Signaling Antagonist II) | MedChemExpress | HY-100535 | ≥95.0% |
| Daphnetin | Selleckchem | S2554 | 99.99% |
| Diacerein | Selleckchem | S4267 | 99.25% |
| Diethyldithiocarbamic acid | Millipore Sigma | 318116 | 97.00% |
| Edelfosine | Millipore Sigma | SML0332 | ≥95% (HPLC) |
| EGFR Inhibitor | Selleckchem | S0360 | 99.56% |
| EGFR Inhibitor III | Millipore Sigma | 324833 | ≥98% by HPLC |
| EGFR/ErbB-2 Inhibitor | Santa Cruz Biotechnology | 179248-61-4 | >97% |
| EKI-785 | Selleckchem | S7557 | 99.08% |
| Epoxomicin | Millipore Sigma | E3652 | ≥95% (HPLC) |

| | | | |
|---|---|---|---|
| Erbstatin analog (Methyl 2,5-dihydroxycinnamate) | Tocris | 0577 | ≥98% (HPLC) |
| Estradiol | Millipore Sigma | 1250008 | NA |
| FGF Receptor Tyrosine Kinase Inhibitor(PD166866) | MedChemExpress | HY-101296 | 99.89% |
| FGF/PDGF/VEGF RTK Inhibitor | Millipore Sigma | 341610 | ≥95% (HPLC) |
| FH535 | MedChemExpress | HY-15721 | 98.72% |
| Forskolin | Millipore Sigma | F6886 | ≥98% (HPLC) |
| GANT61 | Millipore Sigma | G9048 | ≥98% (HPLC) |
| Genistein | Millipore Sigma | G6649 | ≥98% (HPLC) |
| GW 583340 | Tocris | 2239 | ≥97% (HPLC) |
| GW2974 | Millipore Sigma | G0668 | ≥98% (HPLC) |
| Halofuginone | MedChemExpress | HY-N1584 | 99.78% |
| Harpagoside | MedChemExpress | HY-N0396 | 99.41% |
| HDS 029 | Santa Cruz Biotechnology | 881001-19-0 | ≥95% |
| Hedgehog Antagonist VIII | Millipore Sigma | 373402 | ≥95% (HPLC) |
| Herbimycin A | R&D Systems | 1629 | ≥95% |
| Honokiol | R&D Systems | 4590 | ≥98% |
| HU 211 ( Dexanabinol ) | Tocris | 2861 | ≥97% (HPLC) |
| I-OMe-Tyrphostin AG 538 | Millipore Sigma | T7697 | 100.00% |
| IWP-2 | MedChemExpress | HY-13912 | 99.51% |
| IWP-3 | MedChemExpress | HY-100536 | 98.23% |
| IWP-4 | MedChemExpress | HY-12879 | ≥98.0% |
| IWR-1 | MedChemExpress | HY-12238 | 99.49% |
| JAK Inhibitor I (Merck-5, Mk-5) | Millipore Sigma | 420099 | ≥98% by HPLC |
| JAK2 Inhibitor II | Millipore Sigma | 420132 | ≥ 97% by GC |
| JAK2 Inhibitor III, SD-1029 (NSC 371488) | Millipore Sigma | 573098 | ≥80% by HPLC (sum of isomers) |
| JAK2 Inhibitor IV | Millipore Sigma | 420139 | ≥97% (HPLC) |
| JAK2 Inhibitor V, Z3 | Millipore Sigma | 420141 | ≥97% (HPLC) |
| JAK3 Inhibitor I | Millipore Sigma | 420101 | ≥97% (HPLC) |
| JAK3 Inhibitor II (WHI-P154) | Selleckchem | S2867 | 99.77% |
| JAK3 Inhibitor VI | Millipore Sigma | 420126 | ≥98% (HPLC) |
| JAK3 Inhibitor, Negative Control | Santa Cruz Biotechnology | 153437-07-1 | ≥92% |
| Jervine | Selleckchem | S4747 | 98.28% |
| JK 184 | Tocris | 3341 | ≥99% (HPLC) |

| | | | |
|---|---|---|---|
| JNJ 28871063 | Tocris | 3352 | ≥97% (HPLC) |
| JNK Inhibitor II | Millipore Sigma | 420119 | ≥98% (HPLC) |
| Kenpaullone | Selleckchem | S7917 | 99.01% |
| Lactacystin | Millipore Sigma | L6785 | ≥90% (HPLC) |
| Lapatinib ditosylate (GW 572016) | Selleckchem | S1028 | 99.98% |
| Lavendustin A | Tocris | 1331 | ≥98% (HPLC) |
| Lavendustin A methyl ester | Enzo | ALX-350-091-M005 | ≥97% |
| Lavendustin C | Santa Cruz Biotechnology | 125697-93-0 | ≥98% |
| Lavendustin C methyl ester | Enzo | ALX-350-084-M005 | ≥97% |
| Lestaurtinib | Tocris | 3395 | ≥99% (HPLC) |
| Lupeol | Selleckchem | S3614 | 100.00% |
| LY 294002 | Millipore Sigma | 440202 | ≥98% (HPLC) |
| LY2606368 | MedChemExpress | HY-18174 | 98.74% |
| MG 132 | Selleckchem | S2619 | 99.69% |
| MK8776 | Selleckchem | S2735 | 99.98% |
| MPMQ hydrochloride (LY 456236) | Tocris | 2390 | ≥99% (HPLC) |
| Myricetin | Selleckchem | S2326 | 99.79% |
| NF-kB Activation Inhibitor IV | Millipore Sigma | 481412 | ≥98% by HPLC |
| N-Oleoyldopamine | MedChemExpress | HY-108448 | 99.47% |
| NSC109555 | Tocris | 3034 | ≥98% (HPLC) |
| NVP-TAE684 - TAE 684 | Selleckchem | S1108 | 99.73% |
| Panepoxydone | Enzo | ALX-350-109-MC05 | ≥95% (HPLC, MS) |
| PD 153035 (Tyrphostin AG 1517) | Selleckchem | S1079 | 99.26% |
| PD 156273 | Santa Cruz Biotechnology | sc-222136 | NA |
| PD 158780 | Santa Cruz Biotechnology | sc-358789 | ≥98% |
| PD 161570 | Santa Cruz Biotechnology | 192705-80-9 | ≥99% |
| PD 168393 | Santa Cruz Biotechnology | 194423-15-9 | ≥95% |
| PD 173074 | Santa Cruz Biotechnology | 219580-11-7 | ≥98% |
| PD 174265 | Santa Cruz Biotechnology | 216163-53-0 | ≥97% |
| PD 407824 | MedChemExpress | HY-18961 | 98.02% |

| | | | |
|---|---|---|---|
| PDGFR Tyrosine Kinase Inhibitor III | MedChemExpress | HY-112412 | 99.85% |
| PF 477736 | MedChemExpress | HY-10032 | 99.21% |
| PHA739358(Danusertib) | Selleckchem | S1107 | 99.55% |
| Piceatannol | Selleckchem | S3026 | 99.80% |
| Pirfenidone | Selleckchem | S2907 | 99.98% |
| PKCÎ²II/EGFR Inhibitor (CGP 53353) | Tocris | 2442 | ≥99% (HPLC) |
| PKF115-584 (Calphostin C , UCN-1028C) | Tocris | 1626 | ≥95% |
| PNU-74654 | Selleckchem | S8429 | 99.66% |
| PP 3 | Tocris | 2794 | ≥98% (HPLC) |
| PP2 (AG 1879) | Selleckchem | S7008 | 99.50% |
| Prostratin | Millipore Sigma | P0077 | ≥98% (HPLC) |
| Purmorphamine | Millipore Sigma | SML0868 | ≥98% (HPLC) |
| PV 1019 | MedChemExpress | HY-125203 | NA |
| Pyrrolidinedithiocarbamate ammonium | Millipore Sigma | P8765 | ~99% |
| QS11 - Wnt Synergist | Millipore Sigma | 681668 | ≥95% (HPLC) |
| Rapamycin | Selleckchem | S1039 | 99.30% |
| RG-13022 | Selleckchem | S2115 | 99.28% |
| RG-14620 | Selleckchem | S6523 | 99.90% |
| S14-95 | Santa Cruz Biotechnology | sc-222286 | ≥95% |
| SAG | Selleckchem | S7779 | 99.98% |
| SANT-1 | Selleckchem | S7092 | 99.99% |
| SANT-2 | Tocris | 3617 | ≥98% (HPLC) |
| SB 203580 | MedChemExpress | HY-10256 | 99.96% |
| SB 216763 | MedChemExpress | HY-12012 | 99.82% |
| SB 415286 | MedChemExpress | HY-15438 | 99.35% |
| SB-505124 (TGF-β RI Inhibitor III) | Tocris | 3263 | ≥98% (HPLC) |
| SB-525334 (TGF-Î² RI Kinase Inhibitor VIII) | Tocris | 3211 | ≥97% (HPLC) |
| Smad3 Inhibitor, SIS3 | Millipore Sigma | 566405 | ≥90% (HPLC) |
| Solasodine | Millipore Sigma | SML1141 | ≥95% (HPLC) |
| Sorafenib (BAY 43-9006) | MedChemExpress | HY-10201 | 99.92% |
| STA-21 | Selleckchem | S7951 | 99.66% |
| STAT1 Enhancer, 2-NP | MedChemExpress | HY-W013523 | 99.89% |
| STAT3 Inhibitor III, WP1066 | Millipore Sigma | 573097 | ≥97% (HPLC) |

| | | | |
|---|---|---|---|
| STAT3 Inhibitor V, Stattic | MedChemExpress | HY-13818 | 98.92% |
| STAT3 Inhibitor VI, S3I-201 | Millipore Sigma | SML0330 | ≥97% (HPLC) |
| STAT3 Inhibitor VII | Millipore Sigma | 573103 | ≥95% (HPLC) |
| STAT3 Inhibitor VIII, 5,15-DPP (5,15-Diphenylporphyrin ) | Millipore Sigma | 573109 | ≥90% (HPLC) |
| STAT5 Inhibitor | Millipore Sigma | 573108-M | ≥97% (HPLC) |
| SU 11652 | MedChemExpress | HY-112452 | 99.06% |
| SU 4984 | MedChemExpress | HY-118203 | 99.14% |
| SU 5402 | MedChemExpress | HY-10407 | 99.38% |
| SU 5416 | MedChemExpress | HY-10374 | 99.96% |
| SU 6668 | MedChemExpress | HY-10517 | 99.21% |
| Sulindac | MedChemExpress | HY-B0008 | 99.81% |
| Sulindac sulfide | MedChemExpress | HY-B1786 | 98.60% |
| Suramin | Cayman Chemicals | 129-46-4 | ≥98% |
| TAK 165 | Selleckchem | S2216 | 99.94% |
| Tarceva (Erlotininb, CP 358774) | MedChemExpress | HY-50896 | 99.98% |
| TGF-ÃŸ RI Kinase Inhibitor V (SD-208) | MedChemExpress | HY-13227 | 99.41% |
| Tomatidine | MedChemExpress | HY-N2149 | ≥98% |
| Tpl2 Kinase Inhibitor | Millipore Sigma | 616373 | ≥95% (HPLC) |
| Trichodion | Millipore Sigma | IDF00041 | 100.00% |
| Triflusal | MedChemExpress | HY-B0531 | 99.83% |
| Triptolide | MedChemExpress | HY-32735 | 99.86% |
| Tryphostin A8 (AG 10, Tryphostin 63, AG 63) | Santa Cruz Biotechnology | 3785-90-8 | ≥98% |
| Tryphostin AG 494 | MedChemExpress | HY-101042 | 99.31% |
| Tryphostin AG 555 | MedChemExpress | HY-15336 | ≥98% |
| Tryphostin AG 556 | MedChemExpress | HY-101041 | 99.92% |
| Tryphostin AG 825 | MedChemExpress | HY-15844 | 98.02% |
| Tyrphostin 23 (AG 18) | MedChemExpress | HY-15644 | 99.43% |
| Tyrphostin 25 (RG-50875, AG 82) | MedChemExpress | HY-101958 | NA |
| Tyrphostin 46 (AG 99) | MedChemExpress | HY-100962 | 98.36% |
| Tyrphostin 47 (AG 213, RG-50864) | Enzo | BML-EI188-0005 | ≥99% (TLC) |
| Tyrphostin 51 (AG 183) | MedChemExpress | HY-101960A | ≥98.0% |
| Tyrphostin A1 (AG 9) | MedChemExpress | HY-16668 | 99.50% |
| Tyrphostin A9 (AG 17) | Millipore Sigma | T182 | ≥98% (HPLC) |

| | | | |
|---|---|---|---|
| Tyrphostin AG 112 | MedChemExpress | HY-112474 | NA |
| Tyrphostin AG 126 | MedChemExpress | HY-108330 | ≥98.0% |
| Tyrphostin AG 1288 | AdooQ Bioscience | A13587 | >98% |
| Tyrphostin AG 1295 | MedChemExpress | HY-101957 | 99.90% |
| Tyrphostin AG 1296 | MedChemExpress | HY-13894 | 99.25% |
| Tyrphostin AG 1478 | MedChemExpress | HY-13524 | 99.22% |
| Tyrphostin AG 30 | MedChemExpress | HY-118532 | 99.70% |
| Tyrphostin AG 34 | Advance Scientific and Chemicals | T3255 | NA |
| Tyrphostin AG 370 | MedChemExpress | HY-116111 | NA |
| Tyrphostin AG 490 | MedChemExpress | HY-12000 | 99.97% |
| Tyrphostin AG 494 | MedChemExpress | HY-101042 | 99.31% |
| Tyrphostin AG 527 (Tyrphostin B50, Tyrphostin B44, (-) enantiomer) | Santa Cruz Biotechnology | 133550-37-5 | NA |
| Tyrphostin AG 528 | MedChemExpress | HY-100499 | ≥98.0% |
| Tyrphostin AG 537 | Santa Cruz Biotechnology | sc-222388 | ≥98% |
| Tyrphostin AG 658 | MedChemExpress | sc-222390 | ≥98% |
| Tyrphostin AG 698 | Enzo | ALX-270-145-M025 | ≥98% |
| Tyrphostin AG 835 (Tyrphostin B50, Tyrphostin B44, (+) enantiomer) | MedChemExpress | HY-101007A | 97.01% |
| Tyrphostin AG 879 | MedChemExpress | HY-20878 | 99.85% |
| Tyrphostin SU 1498 | MedChemExpress | HY-19326 | 98.01% |
| U 18666A | MedChemExpress | HY-107433 | ≥99.0% |
| Valsartan | MedChemExpress | HY-18204 | 99.87% |
| Vandetanib (ZD 6474) | Selleckchem | S1046 | 99.99% |
| VEGFR Tyrosine Kinase Inhibitor II | Millipore Sigma | 676481 | ≥98% (HPLC) |
| Withaferin A | Selleckchem | S8587 | 99.92% |
| Wnt Agonist | Selleckchem | S8178 | 99.91% |
| ZM 306416 | Selleckchem | S2897 | 99.97% |
| ZM 323881 | Selleckchem | S2896 | 99.90% |
| ZM 323881 | Selleckchem | S2896 | 99.90% |
| ZM 336372 | Selleckchem | S2720 | 99.09% |
| ZM 39923 hydrochloride (JAK3 Inhibitor IV) | Selleckchem | S8004 | 99.92% |
| ZM 447439 | Selleckchem | S1103 | 99.26% |

| | | | |
|---|---|---|---|
| ZM-449829 (JAK3 Inhibitor V) | Cayman Chemicals | 4452-06-6 | ≥95% |

**Supplementary Table 3. Primary hit compounds.**

| Compound | Target(s) & Effect(s) | Reported Function | Fold Change |
|---|---|---|---|
| TAE 684 | ALK Inhibition | Insulin sensitivity | 1.7 |
| Daphnetin | EGFR / PKC inhibition | Insulin secretion & sensitivity | 1.8 |
| Vandetanib (ZD6474) | VEGFR2 / VEGFR3 / EGFR inhibition | Islet cell inflammation, insulin expression, insulin secretion & sensitivity | 1.7 |
| EKI-785 | EGFR inhibition | Insulin secretion & sensitivity | 1.7 |
| BPDQ | EGFR inhibition | Insulin secretion & sensitivity | 1.7 |
| Tyrphostin AG 1478 | EGFR inhibition | Insulin secretion & sensitivity | 2.9 |
| RG-13022 | EGFR inhibition | Insulin secretion & sensitivity | 2.2 |
| CP 358774 | EGFR inhibition | Insulin secretion & sensitivity | 2.3 |
| 6-bromoindirubin-3'-oxime (BIO) | GSK3β inhibition | β cell mass | 2.5 |
| N-Oleoyldopamine | TRPV1 agonist | GLP & substance P secretion | 2.7 |
| Tyrphostin A9 (AG 17) | PDGFR inhibition | β cell proliferation | 3.5 |
| Tyrphostin AG 1295 | PDGFR inhibition | β cell proliferation | 1.8 |
| Tyrphostin AG 1296 | PDGFR inhibition | β cell proliferation | 3.2 |
| Prostratin | PKC activation | Insulin secretion | 2.3 |
| JNJ 28871063 | EGFR / ErbB4 / ErbB2 inhibition | Insulin secretion & sensitivity, β cell proliferation & neogenesis | 2.7 |
| LDN-193189 (DM-3189) | ALK2 / ALK3 inhibition | Insulin secretion, insulin gene expression | 3.0 |
| D4476 | CK1 inhibition | Glucose metabolism | 2.2 |
| Honokiol | Nrf2 / ARE activation | B cell oxidative stress defense & proliferation | 5.3 |
| 2-NP | STAT1 enhancer | Islet inflammation & β cell apoptosis | 1.6 |
| AZD7762 | CHEK1 / 2 inhibition | Not known | 1.8 |
| CCT 241533 | CHEK2 inhibition | Not known | 2.2 |

**Supplementary Table 4. Human Islet Donor Characteristics.**

| Donor No. | Age | Gender | BMI | HbA1c | Disease Status (T2D) |
|---|---|---|---|---|---|
| 1 | 27 | M | 38.7 | 5.2 | No History of Diabetes |
| 2 | 35 | M | 27.52 | 6 | No History of Diabetes |
| 3 | 49 | F | 36.4 | 6 | No History of Diabetes |
| 4 | 31 | M | 36.3 | 5 | No History of Diabetes |
| 5 | 55 | F | 29.7 | 5.6 | No History of Diabetes |
| 6 | 41 | F | 32 | 5.3 | No History of Diabetes |
| 7 | 51 | F | 33 | 5.7 | No History of Diabetes |
| 8 | 37 | F | 24.03 | 5.2 | No History of Diabetes |
| 9 | 37 | M | 31.95 | 5.6 | No History of Diabetes |
| 10 | 44 | M | 25.775 | 6 | No History of Diabetes |
| 11 | 43 | F | 45.49 | 6.7 | T2DM |
| 12 | 50 | F | 30.9 | 5.8 | T2DM |
| 13 | 55 | M | 17.09 | 7 | T2DM |
| 14 | 50 | F | 36 | 6.9 | T2DM |
| 15 | 63 | M | 24 | 6.8 | T2DM |
| 16 | 38 | F | 43.98 | 7 | T2DM |

**Supplementary Table 5. Sequence of sgRNAs.**

| | |
|---|---|
| sgCHEK2_1 | *GTCTCGGGAGTCGGATGTTG* |
| sgCHEK2_2 | *GGCAGCGTTACCCAGTCCCA* |
| scramble sgRNA_1 | *GCTTACAATCGTCGGTCCAAT* |
| scramble sgRNA_1 | *GGTACCATACCGCGTACCCTT* |

**Supplementary Table 6. Kinase Profiling on 1 µM AZD7762 with SelectScreen Biochemical Service by ThermoFisher Scientific**

| Targets | % Inhibition in Kinase Assay | Assay |
|---|---|---|
| ABL1 | 102 | Z'-LYTE |
| ALK | 101 | Z'-LYTE |
| AXL | 96 | Z'-LYTE |
| BMX | 94 | Z'-LYTE |
| BTK | 102 | Z'-LYTE |
| CAMK1 (CaMK1) | 98 | Adapta |
| CHEK1 (CHK1) | 102 | Z'-LYTE |
| CHEK2 (CHK2) | 102 | Z'-LYTE |
| CSF1R (FMS) | 101 | Z'-LYTE |
| EPHA1 | 100 | Z'-LYTE |
| EPHA3 | 79 | LanthaScreen |
| FER | 101 | Z'-LYTE |
| FGR | 101 | Z'-LYTE |
| FLT1 (VEGFR1) | 88 | Z'-LYTE |
| FLT3 | 103 | Z'-LYTE |
| FYN | 101 | Z'-LYTE |
| HCK | 102 | Z'-LYTE |
| IRAK4 | 104 | Z'-LYTE |
| LCK | 96 | Z'-LYTE |
| LYN A | 101 | Z'-LYTE |
| LYN B | 101 | Z'-LYTE |
| MARK1 (MARK) | 100 | Z'-LYTE |
| MKNK2 (MNK2) | 103 | LanthaScreen |
| NUAK1 (ARK5) | 97 | Adapta |
| PAK6 | 75 | Z'-LYTE |
| PDK1 Direct | 107 | Z'-LYTE |
| PLK2 | 12 | Z'-LYTE |
| PRKG2 (PKG2) | 100 | Z'-LYTE |
| PTK6 (Brk) | 94 | Z'-LYTE |
| RET | 101 | Z'-LYTE |
| RPS6KA2 (RSK3) | 98 | Z'-LYTE |
| RPS6KA3 (RSK2) | 93 | Z'-LYTE |
| SIK1 | 99 | LanthaScreen |
| STK3 (MST2) | 101 | Z'-LYTE |
| STK4 (MST1) | 101 | Z'-LYTE |
| SYK | 100 | Z'-LYTE |
| YES1 | 100 | Z'-LYTE |

**Supplementary Table 7. Raw data of kinase Profiling on 1 µM AZD7762 with SelectScreen Biochemical Service by ThermoFisher Scientific.**

**Z'-LYTE**

| **[ATP] Tested** | **Kinase Tested** | **% Inhibition** | | **Difference Between Data Points** | **Development Reaction Interference** | **Test Compound Interference** | | **Z'** | **Kinase Part# / Lot#** |
|---|---|---|---|---|---|---|---|---|---|
| (µM) | | Point 1 | Point 2 | ⎪Point 1 - Point 2⎪ | | Coumarin | Fluorescein | | |
| Km app | ABL1 | 102 | 102 | 0 | **Pass** | **Pass** | **Pass** | **0.70** | P3049/1913217 |
| Km app | ALK | 101 | 101 | 0 | **Pass** | **Pass** | **Pass** | **0.84** | PV3867/1542512 |
| Km app | AXL | 96 | 96 | 0 | **Pass** | **Pass** | **Pass** | **0.92** | A31515/1827503 |
| Km app | BMX | 93 | 95 | 2 | **Pass** | **Pass** | **Pass** | **0.69** | PV3371/1992619 |
| Km app | BTK | 102 | 102 | 0 | **Pass** | **Pass** | **Pass** | **0.85** | PV3363/2279587 |
| Km app | CHEK1 | 103 | 101 | 2 | **Pass** | **Pass** | **Pass** | **0.81** | P3040/2238473 |
| Km app | CHEK2 | 100 | 105 | 4 | **Pass** | **Pass** | **Pass** | **0.70** | PV3367/1990123 |
| Km app | CSF1R | 102 | 99 | 2 | **Pass** | **Pass** | **Pass** | **0.78** | PV3249/2112355 |
| Km app | EPHA1 | 99 | 100 | 1 | **Pass** | **Pass** | **Pass** | **0.77** | PV3841/1924904 |
| Km app | FER | 101 | 101 | 0 | **Pass** | **Pass** | **Pass** | **0.82** | PV3806/1871897 |
| Km app | FGR | 101 | 101 | 0 | **Pass** | **Pass** | **Pass** | **0.90** | P3041/26670 |
| Km app | FLT1 | 86 | 89 | 3 | **Pass** | **Pass** | **Pass** | **0.77** | PV3666/1912726 |
| Km app | FLT3 | 103 | 103 | 0 | **Pass** | **Pass** | **Pass** | **0.84** | PV3182/2001422 |
| Km app | FYN | 102 | 101 | 0 | **Pass** | **Pass** | **Pass** | **0.86** | P3042/1912011 |
| Km app | HCK | 102 | 103 | 1 | **Pass** | **Pass** | **Pass** | **0.83** | PV6128/2071130 |

| | | | | | | | | | |
|---|---|---|---|---|---|---|---|---|---|
| Km app | IRAK4 | 105 | 102 | 3 | **Pass** | **Pass** | **Pass** | **0.74** | PV3362/2066052 |
| Km app | LCK | 96 | 96 | 1 | **Pass** | **Pass** | **Pass** | **0.83** | P3043/2101210 |
| Km app | LYN A | 102 | 100 | 2 | **Pass** | **Pass** | **Pass** | **0.89** | PV6448/2022332 |
| Km app | LYN B | 99 | 102 | 3 | **Pass** | **Pass** | **Pass** | **0.83** | P2907/21076 |
| Km app | MARK1 | 101 | 98 | 2 | **Pass** | **Pass** | **Pass** | **0.78** | PV4395/2168227 |
| Km app | PAK6 | 79 | 72 | 7 | **Pass** | **Pass** | **Pass** | **0.77** | PV3502/1885919 |
| Km app | PDK1 | 106 | 109 | 3 | **Pass** | **Pass** | **Pass** | **0.74** | P3001/1933240 |
| Km app | PLK2 | 13 | 10 | 3 | **Pass** | **Pass** | **Pass** | **0.83** | PV4204/2047482 |
| Km app | PRKG2 | 99 | 101 | 2 | **Pass** | **Pass** | **Pass** | **0.77** | PV3973/1893849 |
| Km app | PTK6 | 94 | 94 | 1 | **Pass** | **Pass** | **Pass** | **0.85** | PV3291/1901151 |
| Km app | RET | 100 | 101 | 1 | **Pass** | **Pass** | **Pass** | **0.82** | PV3819/1706568 |
| Km app | RPS6KA2 | 97 | 98 | 0 | **Pass** | **Pass** | **Pass** | **0.91** | A31517/71600 |
| Km app | RPS6KA3 | 93 | 92 | 1 | **Pass** | **Pass** | **Pass** | **0.84** | PV3323/1571417 |
| Km app | STK3 | 103 | 99 | 4 | **Pass** | **Pass** | **Pass** | **0.76** | PV4805/1767304 |
| Km app | STK4 | 101 | 100 | 1 | **Pass** | **Pass** | **Pass** | **0.74** | PV3854/2050660 |
| Km app | SYK | 101 | 99 | 2 | **Pass** | **Pass** | **Pass** | **0.85** | PV3857/1885916 |
| Km app | YES1 | 99 | 101 | 2 | **Pass** | **Pass** | **Pass** | **0.90** | A15557/50645 |

## Adapta

| **[ATP] Tested** | **Kinase Tested** | **% Inhibition** | **Difference Between Data Points** | **Test Compound Interference** | **Z'** | **Kinase Part# / Lot#** |
|---|---|---|---|---|---|---|

| | | | | | | | | |
|---|---|---|---|---|---|---|---|---|
| (µM) | | Point 1 | Point 2 | ⎜Point 1 - Point 2⎜ | Donor | Acceptor | | |
| 100 | CAMK1 | 98 | 99 | 1 | **Pass** | **Pass** | **0.84** | PV4391/1703731 |
| Km app | NUAK1 | 95 | 98 | 3 | **Pass** | **Pass** | **0.83** | PV4127/2051364 |

**LanthaScreen Binding**

| **Kinase Tested** | **% Displacement** | | **Difference Between Data Points** | **Test Compound Interference** | | **Z'** | **Kinase Part# / Lot#** |
|---|---|---|---|---|---|---|---|
| | Point 1 | Point 2 | ⎜Point 1 - Point 2⎜ | Donor | Acceptor | | |
| EPHA3 | 76 | 83 | 6 | **Pass** | **Pass** | **0.81** | PV3359/673524 |
| MKNK2 (MNK2) | 107 | 99 | 7 | **Pass** | **Pass** | **0.70** | PV5607/1793520 |
| SIK1 | 99 | 100 | 1 | **Pass** | **Pass** | **0.87** | PV6445/70550 |

**Supplementary Table 8. Sequences of shRNAs.**

| | |
|---|---|
| shABL1_1 | *CCTCAGTTCGGTGAAGGAAAT* |
| shABL1_2 | *CCGCCTTCATCCCTCTCATAT* |
| shALK_1 | *GCAGAATACAGCACCCAAATC* |
| shALK_2 | *GTGGAGCCACCTACGTATTTA* |
| shAXL_1 | *GCGGTCTGCATGAAGGAATTT* |
| shAXL_2 | *CCTAAGCATCTAAGTTATAAG* |
| shBMX_1 | *GTGTTCTCTGTATTGTCTATT* |
| shBMX_2 | *GAGTGCTGATAAGAATGAATA* |
| shBTK_1 | *CTCATATCCAGGCTCAAATAT* |
| shBTK_2 | *GCGGAAGGGTGATGAATATTT* |
| shCAMK1_1 | *GAAGGCCGAGTACGAGTTTGA* |
| shCAMK1_2 | *CCAAACTCTTTGAACAGATTT* |
| shCHEK1_1 | *GCAACAGTATTTCGGTATAAT* |
| shCHEK1_2 | *GTGACAGCTGTCAGGAGTATT* |
| shCSF1R_1 | *GTGAACAGCAAGTTCTATAAA* |
| shCSF1R_2 | *ACAGGAGAGAGCGGGACTATA* |
| shEPHA1_1 | *CAGTTTAGCCACCCGCATATT* |
| shEPHA1_2 | *TGTGGCCATTAAGACCTTAAA* |
| shEPHA3_1 | *CAAACTATCTGGCCATATTTA* |
| shEPHA3_2 | *GCTGATATGATCTTGAGTATA* |
| shFER_1 | *CAGAACAACTTAGTAGGATAA* |
| shFER_2 | *CCACCTCCAGTAGTAAATTAT* |
| shFGR_1 | *CCCTGTTCATTGCCCTGTATG* |
| shFGR_2 | *GAACGCATGAACTACATTCAC* |
| shFLT1_1 | *ACTCGTGGCTACTCGTTAATT* |
| shFLT1_2 | *CGCCGGAAGTTGTATGGTTAA* |
| shFLT3_1 | *CCAATTCAAGTGAAGATTATG* |
| shFLT3_2 | *GGTGTCGAGCAGTACTCTAAA* |
| shFYN_1 | *CATCGAGCGCATGAATTATAT* |
| shFYN_2 | *GGTTACATTCCCAGCAATTAT* |
| shG6PD_1 | *GGATACACACATATTCATCATC* |
| shG6PD_2 | *TTGACCTCAGCTGCACATT* |
| shHCK_1 | *GGGAGATACCGTGAAACATTA* |
| shHCK_2 | *CGTGGTTGCCCTGTATGATTA* |
| shIRAK4_1 | *CTAATAACATTGGGCTAATAT* |

| | |
|---|---|
| shIRAK4_2 | *CCTCTGCTTAGTATATGTTTA* |
| shLCK_1 | *GCATGAACTGGTCCGCCATTA* |
| shLCK_2 | *GCCATTAACTACGGGACATTC* |
| shLuc | *CGTACGCGGAATACTTCGA* |
| shLYN_1 | *AGACTCAACCAGTACGTAATA* |
| shLYN_2 | *GGAATCCTCCTATACGAAATT* |
| shMARK1_1 | *ATTTCGAGAAGTACGAATAAT* |
| shMARK1_2 | *CGACGCAGCGTTGCTTATAAT* |
| shMKNK2_1 | *CGCCGTCAAGATCATTGAGAA* |
| shMKNK2_2 | *CCTGGGCGTCATCTTGTATAT* |
| shNUAK1_1 | *TGGCCGAGTGGTTGCTATAAA* |
| shNUAK1_2 | *GCTCGATGACAACTGCAATAT* |
| shPAK6_1 | *GACCCTTTCTATCTAGATAAT* |
| shPAK6_2 | *GTGTTACCTTTCAAGTTTATC* |
| shPDK1_1 | *CAGATGCAGTTATCTACATTA* |
| shPDK1_2 | *GAAGTAGAAGTCTACCATATT* |
| shPLK1_1 | *GTTCTTTACTTCTGGCTATAT* |
| shPLK1_2 | *GATACTACCTACGGCAAATT* |
| shPP2A _1 | *ACCGGAATGTAGTAACGATT* |
| shPP2A_2 | *GCTAGTGATGGAGGGATATAA* |
| shPPP2CB_1 | *GCGAGAAGGCAAAGGAAATTT* |
| shPPP2CB_2 | *CTATGTAGACAGAGGATATTA* |
| shPRKG2_1 | *ATGCTGAGGGTTACCTTAAAT* |
| shPRKG2_2 | *AGCTATCAGGCTGGGATAAAG* |
| shPTK6_1 | *AGGCCATTACTCCACCAAATC* |
| shPTK6_2 | *ACCTCTCCCATGACCACAATA* |
| shRET_1 | *CCGCTGGTGGACTGTAATAAT* |
| shRET_2 | *GGGCGACCGTACATGACTATA* |
| shRPS6KA2_1 | *TCGATATCTGACGCAGCTAAA* |
| shRPS6KA2_2 | *TCTATGATGATGGCAAGTTTG* |
| shRPS6KA3_1 | *CCGTAATTGCTTAGGAGTATT* |
| shRPS6KA3_2 | *GTATTAGGGCAGGGATCATTT* |
| shSIK1_1 | *GCGCGTGCATTGATTACTATC* |
| shSIK1_2 | *ACGATTAGATTCAAGCAATTT* |
| shSTK3_1 | *CCCACTGCTAAGGAGTTATTG* |
| shSTK3_2 | *GCAGGGTTTGTCATTAATAAT* |
| shSTK4_1 | *GTTCTGTATCTGATATCATTC* |

| | |
|---|---|
| shSTK4_2 | *GCCCTCATGTAGTCAAATATT* |
| shSYK_1 | *GCATGAGTGATGGGCTTTATT* |
| shSYK_2 | *GCATGAGTGATGGGCTTTATT* |
| shYES1_1 | *CAGGTGGTGTTACTATATTTG* |
| shYES1_2 | *GCTGATGGTATGGCATATATT* |

**Supplementary Table 9. Quantitative RT-PCR Primers.**

| Gene | Forward Primer | Reverse Primer |
|---|---|---|
| *ABL1* | *AAGCCGCTCGTTGGAACTC* | *AGACCCGGAGCTTTTCACCT* |
| *ALK* | *TCTCATCGCAGCCGATATGG* | *GGCATCTCCTTAGAACGCTCT* |
| *AXL* | *GTGGGCAACCCAGGGAATATC* | *GTACTGTCCCGTGTCGGAAAG* |
| *BMX* | *GAGCAGACGCCTGTAGAGAGA* | *ACCCACTATGGTACTTGACCAG* |
| *BTK* | *TCTGAAGCGATCCCAACAGAA* | *TGCACGGTCAAGAGAAACAGG* |
| *CAMK1* | *CGACTTCCGAGATGTTCTGGG* | *ATCTTGTGCAGGACAGCAATC* |
| *CHEK1* | *ATATGAAGCGTGCCGTAGACT* | *TGCCTATGTCTGGCTCTATTCTG* |
| *CSF1R* | *GGGAATCCCAGTGATAGAGCC* | *TTGGAAGGTAGCGTTGTTGGT* |
| *EPHA1* | *AGATGGGTGGAGTGAACAGC* | *CCTTGCATTGGGCAGTCCT* |
| *EPHA3* | *ACTCTACGAGACTGCAATAGCA* | *TCCCCAAGATCCATTTGAGTGA* |
| *FER* | *ACTCTGGACCTTTACACAGGC* | *GCTTTGTCGTATCGTTCCTTGG* |
| *FGR* | *GGGCAGCAGACCACTATGG* | *CCAGGGTTGATGGCCTGAG* |
| *FLT1* | *TTTGCCTGAAATGGTGAGTAAGG* | *TGGTTTGCTTGAGCTGTGTTC* |
| *FLT3* | *AGGGACAGTGTACGAAGCTG* | *GCTGTGCTTAAAGACCCAGAG* |
| *FYN* | *ATGGGCTGTGTGCAATGTAAG* | *GAAGCTGGGGTAGTGCTGAG* |
| *G6PD* | *ACGACGAAGCGCAGACAG* | *TCCGACTGATGGAAGGCATC* |
| *HCK* | *CCCTGTATGATTACGAGGCCA* | *CACTCCCCGGATTCCTCTAGG* |
| *IRAK4* | *CCTGACTCCTCAAGTCCAGAA* | *ACAGAAATGGGTCGTTCATCAAA* |
| *LCK* | *TGCCATTATCCCATAGTCCCA* | *GAGCCTTCGTAGGTAACCAGT* |
| *LYN* | *TGCAGAGGGAATGGCATACAT* | *TGACTCGGAGACCAGAACATTAG* |
| *MARK1* | *GAGCGGGACACGGAAAATCAT* | *TGCTACTCGACTTGGTAGGCT* |
| *MKNK2* | *CCAGCCGAACTTCAGGGTTT* | *CGTCCGGGATGTCAATGGG* |
| *NUAK1* | *AAGGCACCTACGGCAAAGTC* | *GTCTGATGTGAACCATGTCTTGT* |
| *PAK6* | *ACCAATAGGCATGGAATGAAGG* | *GCGGTCGGAAAGAGGAGTTG* |
| *PDK1* | *CTGTGATACGGATCAGAAACCG* | *TCCACCAAACAATAAAGAGTGCT* |
| *PLK1* | *CCATCACCTGCCTGACCATT* | *GGGGGTTCTCCAAGCCTTTA* |
| *PPP2CA* | *ATCCCCCAAAGGCAGTCTTG* | *TTGTCAGGATTTCTTTAGCCTTGA* |
| *PRKG2* | *TTTGGGGAGCTTGCCATTTTA* | *ATACCTCTCGATCTAGTGCCC* |
| *PTK6* | *TGCCCCATTGGGATGACTG* | *GTACAGCGCCAGGATGTGTTT* |
| *RET* | *GTGTCTTCGATGCAGACGTG* | *CATGGTGCGGTTCTCCGAG* |
| *RPS6KA2* | *GAAATTAAGCGCCATCCCTTCT* | *CCACTGCTGGTTTGAACGGT* |
| *RPS6KA3* | *CGCTGAGAATGGACAGCAAAT* | *TCCAAATGATCCCTGCCCTAAT* |
| *SIK1* | *CTCCGGGTGGGTTTTTACGAC* | *CTGCGTTTTGGTGACTCGATG* |
| *STK3* | *CTTTGGTCCGATGATTTCACCG* | *GGATGCTGTAAAAGTTGTGTTGC* |

| | | |
|---|---|---|
| *STK4* | *CCTCCCACATTCCGAAAACCA* | *GCACTCCTGACAAATGGGTG* |
| *SYK* | *GTGTCATTCAATCCGTATGAGCC* | *TTTCGGTCCAGGTAAACCTCC* |
| *YES1* | *GGAAGCAAGATCAATCGCTACA* | *TCACCCCTTATCTCATCCCAAT* |

**Supplementary Table 10. Numbers of mice and islets analyzed in Extended Data Figures 5b, 5d, 5e, 5g, 5h and 5j.**

| Figure | Marker | # of mice | # of islets |
|---|---|---|---|
| Extended Data Fig. 5b | INS | WT_1 | 10 |
| | | WT_2 | 10 |
| | | WT_3 | 11 |
| | | *Chk2*$^{-/-}$_1 | 7 |
| | | *Chk2*$^{-/-}$_2 | 7 |
| | | *Chk2*$^{-/-}$_3 | 8 |
| Extended Data Fig. 5d | INS | WT_1 | 18 |
| | | WT_2 | 15 |
| | | WT_3 | 16 |
| | | WT_4 | 13 |
| | | *Chk2*$^{-/-}$_1 | 29 |
| | | *Chk2*$^{-/-}$_2 | 27 |
| | | *Chk2*$^{-/-}$_3 | 28 |
| | | *Chk2*$^{-/-}$_4 | 25 |
| | | *Chk2*$^{-/-}$_5 | 31 |
| | | *Chk2*$^{-/-}$_6 | 30 |
| | | *Chk2*$^{-/-}$_7 | 27 |
| Extended Data Fig. 5e | GCG | WT_1 | 7 |
| | | WT_2 | 6 |
| | | WT_3 | 8 |
| | | WT_4 | 7 |
| | | *Chk2*$^{-/-}$_1 | 6 |
| | | *Chk2*$^{-/-}$_2 | 6 |
| | | *Chk2*$^{-/-}$_3 | 5 |
| | | *Chk2*$^{-/-}$_4 | 6 |
| Extended Data Fig. 5g | SST | WT_1 | 15 |
| | | WT_2 | 12 |
| | | WT_3 | 15 |
| | | WT_4 | 15 |
| | | WT_5 | 14 |
| | | WT_6 | 16 |
| | | *Chk2*$^{-/-}$_1 | 27 |
| | | *Chk2*$^{-/-}$_2 | 13 |
| | | *Chk2*$^{-/-}$_3 | 29 |

| | | | |
|---|---|---|---|
| | | *Chk2*$^{-/-}$_4 | 30 |
| | | *Chk2*$^{-/-}$_5 | 28 |
| | | *Chk2*$^{-/-}$_6 | 22 |
| | | *Chk2*$^{-/-}$_7 | 30 |
| | | *Chk2*$^{-/-}$_8 | 27 |
| Extended Data Fig. 5h | PP | WT_1 | 18 |
| | | WT_2 | 17 |
| | | WT_3 | 18 |
| | | WT_4 | 14 |
| | | WT_5 | 18 |
| | | *Chk2*$^{-/-}$_1 | 27 |
| | | *Chk2*$^{-/-}$_2 | 29 |
| | | *Chk2*$^{-/-}$_3 | 30 |
| | | *Chk2*$^{-/-}$_4 | 28 |
| | | *Chk2*$^{-/-}$_5 | 22 |
| | | *Chk2*$^{-/-}$_6 | 30 |
| | | *Chk2*$^{-/-}$_7 | 28 |
| Extended Data Fig. 5j | NGN3 | WT_1 | 5 |
| | | WT_2 | 5 |
| | | WT_3 | 6 |
| | | WT_4 | 6 |
| | | WT_5 | 5 |
| | | *Chk2*$^{-/-}$_1 | 5 |
| | | *Chk2*$^{-/-}$_2 | 10 |
| | | *Chk2*$^{-/-}$_3 | 5 |
| | | *Chk2*$^{-/-}$_4 | 5 |
| | | *Chk2*$^{-/-}$_5 | 5 |
| | | *Chk2*$^{-/-}$_6 | 8 |
