# Supplementary material for: Checkpoint kinase 2 controls insulin secretion and glucose homeostasis
Source: Nat Chem Biol. 2023 Nov 9;20(5):566–76. doi: 10.1038/s41589-023-01466-4 (PMC11062908; doi:10.1038/s41589-023-01466-4)
Supplement: Supplementary file 2 — Reporting Summary [file 41589_2023_1466_MOESM2_ESM.pdf]

nature portfolio

Corresponding author(s): Shuibing Chen, Chengyang Liu

Last updated by author(s): Sep 10, 2023

Reporting Summary

Nature Portfolio wishes to improve the reproducibility of the work that we publish. This form provides structure for consistency and transparency in reporting. For further information on Nature Portfolio policies, see our Editorial Policies and the Editorial Policy Checklist.

Statistics

For all statistical analyses, confirm that the following items are present in the figure legend, table legend, main text, or Methods section.

n/a	Confirmed	
☐	☒	The exact sample size (*n*) for each experimental group/condition, given as a discrete number and unit of measurement
☐	☒	A statement on whether measurements were taken from distinct samples or whether the same sample was measured repeatedly
☐	☒	The statistical test(s) used AND whether they are one- or two-sided<br>*Only common tests should be described solely by name; describe more complex techniques in the Methods section.*
☐	☒	A description of all covariates tested
☒	☐	A description of any assumptions or corrections, such as tests of normality and adjustment for multiple comparisons
☐	☒	A full description of the statistical parameters including central tendency (e.g. means) or other basic estimates (e.g. regression coefficient) AND variation (e.g. standard deviation) or associated estimates of uncertainty (e.g. confidence intervals)
☐	☒	For null hypothesis testing, the test statistic (e.g. *F*, *t*, *r*) with confidence intervals, effect sizes, degrees of freedom and *P* value noted<br>*Give P values as exact values whenever suitable.*
☒	☐	For Bayesian analysis, information on the choice of priors and Markov chain Monte Carlo settings
☒	☐	For hierarchical and complex designs, identification of the appropriate level for tests and full reporting of outcomes
☐	☒	Estimates of effect sizes (e.g. Cohen's *d*, Pearson's *r*), indicating how they were calculated

Our web collection on statistics for biologists contains articles on many of the points above.

Software and code

Policy information about availability of computer code

Data collection	no software was used
Data analysis	Graphpad Prism 9 Graphpad software https://www.graphpad.com<br>Image Studio software 5.2.5 https://www.licor.com/bio/image-studio-lite/download<br>R Studios 2021.09.0 https://www.rstudio.com/<br>R 4.1.2 https://cran.r-project.org/<br>pheatmap 1.0.12 https://www.rdocumentation.org/packages/pheatmap/versions/1.0.12/topics/pheatmap<br>QIAGEN Ingenuity Pathway Analysis (QIAGEN IPA) September 2021 https://digitalinsights.qiagen.com/products-overview/discovery-insights-portfolio/analysis-and-visualization/qiagen-ipa/

For manuscripts utilizing custom algorithms or software that are central to the research but not yet described in published literature, software must be made available to editors and reviewers. We strongly encourage code deposition in a community repository (e.g. GitHub). See the Nature Portfolio guidelines for submitting code & software for further information.

Data

Policy information about availability of data

All manuscripts must include a data availability statement. This statement should provide the following information, where applicable:

- Accession codes, unique identifiers, or web links for publicly available datasets
- A description of any restrictions on data availability
- For clinical datasets or third party data, please ensure that the statement adheres to our policy

All data needed to evaluate the conclusions in the paper are present in the paper and/or the Supplementary Materials. Additional data related to this paper are

uploaded to Mendeley Data Repository DOI: 10.17632/n4bssm8kww.1. For bulk RNA seq experiment, to measure gene expression, the trimmed reads were aligned to the human reference genome (GRCh37). The raw RNA-seq data have been deposited in the Gene Expression Omnibus (GEO) database under accession number GSE239335.

Field-specific reporting

Please select the one below that is the best fit for your research. If you are not sure, read the appropriate sections before making your selection.

☒ Life sciences ☐ Behavioural & social sciences ☐ Ecological, evolutionary & environmental sciences

For a reference copy of the document with all sections, see nature.com/documents/nr-reporting-summary-flat.pdf

Life sciences study design

All studies must disclose on these points even when the disclosure is negative.

Sample size	No sample size calculation was performed. Sample size for animal experiments was determined based on criteria set by IACUC. Our sample sizes were chosen based on a combination of prior literature, practical considerations, and preliminary data analyses.
Data exclusions	no data were excluded
Replication	All experiments were successfully repeated for at least 3 times.
Randomization	Animals were randomly assigned into treatment groups. For non-animal experiments, samples were randomly assigned to experimental groups.
Blinding	Investigators were blinded during data collection and analysis.

Reporting for specific materials, systems and methods

We require information from authors about some types of materials, experimental systems and methods used in many studies. Here, indicate whether each material, system or method listed is relevant to your study. If you are not sure if a list item applies to your research, read the appropriate section before selecting a response.

Materials & experimental systems

n/a	Involved in the study	
☐	☒	Antibodies
☐	☒	Eukaryotic cell lines
☒	☐	Palaeontology and archaeology
☐	☒	Animals and other organisms
☒	☐	Human research participants
☒	☐	Clinical data
☒	☐	Dual use research of concern

Methods

n/a	Involved in the study	
☒	☐	ChIP-seq
☒	☐	Flow cytometry
☒	☐	MRI-based neuroimaging

Antibodies

Antibodies used	mouse monoclonal anti-β-Actin, Invitrogen, MA1-140, 1:20,000 https://www.thermofisher.com/antibody/product/beta-Actin-Antibody-clone-15G5A11-E2-Monoclonal/MA1-140<br>goat anti-insulin antibody, Dako, Agilent, IR002, 1:50, https://www.agilent.com/en/product/immunohistochemistry/antibodies-controls/primary-antibodies/insulin-%28autostainer-link-48%29-76277#specifications<br>mouse monoclonal anti-CHEK2, Cell Signaling, 3440T, 1:1,000 https://www.cellsignal.com/products/primary-antibodies/chk2-1c12-mouse-mab/3440<br>rabbit monoclonal anti-phospho CHEK2 T68, Cell Signaling, 2197S (C13C1), 1:1,000 https://www.cellsignal.com/products/primary-antibodies/phospho-chk2-thr68-c13c1-rabbit-mab/2197<br>rabbit anti-eIF2a antibody, Cell Signaling #5324(D7D3), 1:1000, https://www.cellsignal.com/products/primary-antibodies/eif2a-d7d3-xp-rabbit-mab/5324?_requestid=483558<br>rabbit anti-phospho eIF2a Ser51 antibody, Cell Signaling #3597 (119A11), 1:1000, https://www.cellsignal.com/product/productDetail.jsp?productId=3597<br>rabbit anti-α-Tubulin polyclonal Antibody, Cell Signaling #2144, 1:1000, https://www.cellsignal.com/product/productDetail.jsp?productId=2144<br>rabbit anti-phospho PLK1 T210, Abcam ab155095 (EPNCIR167), 1:200, https://www.abcam.com/products/primary-antibodies/plk1-phospho-t210-antibody-epncir167-ab155095.html<br>mouse anti-PLK1, Abcam ab17056 (35-206), 1:1000, https://www.abcam.com/products/primary-antibodies/plk1-antibody-35-206-ab17056.html<br>sheep anti-neurogenin 3 antibody, R&D AF3444, 1:200, ,https://www.rndsystems.com/products/human-neurogenin-3-antibody_af3444?

gclid=CjwKCAjwvdajBhBEEiwAeMh1U1_q_EvU39cQygwHM3jQL4NLb6fev93jm9kj58AFnxkgiTMTdXECWxoCIAIQAvD_BwE&gclsrc=aw.ds
rat anti-somatostatin antibody, R&D MAB2358 (906552), 1:100, https://www.rndsystems.com/products/human-mouse-somatostatin-antibody-906552_mab2358?gclid=CjwKCAjwvdajBhBEEiwAeMh1U-vj2X2W8tauQ5A12bA23jKe7gOszzQiImvJ9uSNb8c6NNOe3px1JBoCQ-oQAvD_BwE&gclsrc=aw.ds
rabbit anti-glucagon antibody, cell signaling 2760, 1:200, https://www.cellsignal.com/products/primary-antibodies/glucagon-antibody/2760
goat anti-polypeptide Y antibody, Novus NB-100-1793, 1:200, https://www.novusbio.com/products/pancreatic-polypeptide-pp-antibody_nb100-1793
rabbit anti-Ki67 antibody, Abcam, ab15580, 1:500, https://www.abcam.com/products/primary-antibodies/ki67-antibody-ab15580.html
IRDye® 800CW Goat anti-Rabbit IgG Secondary Antibody https://www.licor.com/bio/reagents/irdye-800cw-goat-anti-rabbit-igg-secondary-antibody
IRDye® 680RD Donkey anti-Mouse IgG Secondary Antibody https://www.licor.com/bio/reagents/irdye-680rd-donkey-anti-mouse-igg-secondary-antibody

Validation

mouse monoclonal anti-β-Actin, Invitrogen, MA1-140
validated in SMCC-7721 human hepatocellular carcinoma cells for western blot
Oncology Reports 2018 - CRISPR/Cas9-mediated hypoxia inducible factor-1α knockout enhances the antitumor effect of transarterial embolization in hepatocellular carcinoma. Dilution 1:2000

mouse monoclonal anti-CHEK2, Cell Signaling, 3440T
validated by manufacturer in human HeLa cells for western blot at 1:1000
Western blot analysis of extracts from control HeLa cells (lane 1) or Chk2 knockout HeLa cells (lane 2) using Chk2 (1C12) Mouse mAb (upper) or α-Actinin (D6F6) XP® Rabbit mAb #6487 (lower). The absence of signal in the Chk2 knockout HeLa cells confirms the specificity of the antibody for Chk2.

rabbit monoclonal anti-phospho CHEK2 T68, Cell Signaling, 2197S
validated by manufacturer in human HeLa cells for western blot at 1:1000
Western blot analysis of extracts from HeLa cells, untreated or UV-treated, using Phospho-Chk2 (Thr68) (C13C1) Rabbit mAb.

goat anti-insulin antibody, Dako, Agilent, IR002, 1:50
https://www.agilent.com/en/product/immunohistochemistry/antibodies-controls/primary-antibodies/insulin-%28autostainer-link-48%29-76277#specifications

sheep anti-neurogenin 3 antibody, R&D AF3444, 1:200
https://www.rndsystems.com/products/human-neurogenin-3-antibody_af3444?gclid=CjwKCAjwvdajBhBEEiwAeMh1U1_q_EvU39cQygwHM3jQL4NLb6fev93jm9kj58AFnxkgiTMTdXECWxoCIAIQAvD_BwE&gclsrc=aw.ds

rat anti-somatostatin antibody, R&D MAB2358, 1:100
https://www.rndsystems.com/products/human-mouse-somatostatin-antibody-906552_mab2358?gclid=CjwKCAjwvdajBhBEEiwAeMh1U-vj2X2W8tauQ5A12bA23jKe7gOszzQiImvJ9uSNb8c6NNOe3px1JBoCQ-oQAvD_BwE&gclsrc=aw.ds

rabbit anti-glucagon antibody, cell signaling 2760, 1:200
https://www.cellsignal.com/products/primary-antibodies/glucagon-antibody/2760

goat anti-polypeptide Y antibody, Novus NB-100-1793, 1:200
https://www.novusbio.com/products/pancreatic-polypeptide-pp-antibody_nb100-1793

rabbit anti-Ki67 antibody, Abcam, ab15580, 1:500
https://www.abcam.com/products/primary-antibodies/ki67-antibody-ab15580.html

rabbit anti-eIF2a antibody, Cell Signaling #5324, 1:1000
https://www.cellsignal.com/products/primary-antibodies/eif2a-d7d3-xp-rabbit-mab/5324

rabbit anti-phospho eIF2a Ser51 antibody, Cell Signaling #3597, 1:1000
https://www.cellsignal.com/products/primary-antibodies/phospho-eif2a-ser51-119a11-rabbit-mab/3597

rabbit anti-α-Tubulin Antibody Cell Signaling #2144, 1:1000
https://www.cellsignal.com/products/primary-antibodies/a-tubulin-antibody/2144

rabbit anti-phospho PLK1 T210, Abcam ab155095, 1:200
https://www.abcam.com/products/primary-antibodies/plk1-phospho-t210-antibody-epncir167-ab155095.html

mouse anti-PLK1, Abcam ab17056, 1:1000
https://www.abcam.com/products/primary-antibodies/plk1-antibody-35-206-ab17056.html

Eukaryotic cell lines

Policy information about cell lines

Cell line source(s)	Dr. Mingming Hao (MIN6 cells), Dr. Raphael Scharfmann (EndoC-βH1 cells), Dr. Philippe Ravassard (EndoC-βH1 cells), HEK293T cells (ATCC, CRL_3216)
Authentication	Cell lines were authenticated by glucose-stimulated insulin secretion test.
Mycoplasma contamination	All cell lines were tested negative for mycoplasma contamination.
Commonly misidentified lines (See ICLAC register)	No commonly misidentified cell lines were used in the study.

Animals and other organisms

Policy information about studies involving animals; ARRIVE guidelines recommended for reporting animal research

Laboratory animals	Cynomolgus macaques (male, 11 & 12 year-old), C57BL/6J mice (male, 8 week-old), B6.Cg-Lepob/J mice (16 week-old), C57BL/6 N-Atm1BrdChek2tm1b(EUCOMM)Hmgu/JMmucd mice (male & female, 8 month-old), CD-1/ICR mice (male, 8-12 weeks)
Wild animals	The study did not involve wild animals.
Field-collected samples	The study did not involve field-collected samples.
Ethics oversight	IACUC at Weill Cornell Medicine & University of Pennsylvania.

Note that full information on the approval of the study protocol must also be provided in the manuscript.
