# Supplementary material for: Checkpoint kinase 2 controls insulin secretion and glucose homeostasis
Source: Nat Chem Biol. 2023 Nov 9;20(5):566–76. doi: 10.1038/s41589-023-01466-4 (PMC11062908; doi:10.1038/s41589-023-01466-4)
Supplement: Supplementary file 7 — Unmodified blots. [file 41589_2023_1466_MOESM7_ESM.pdf]

**Source Data Figure 4**

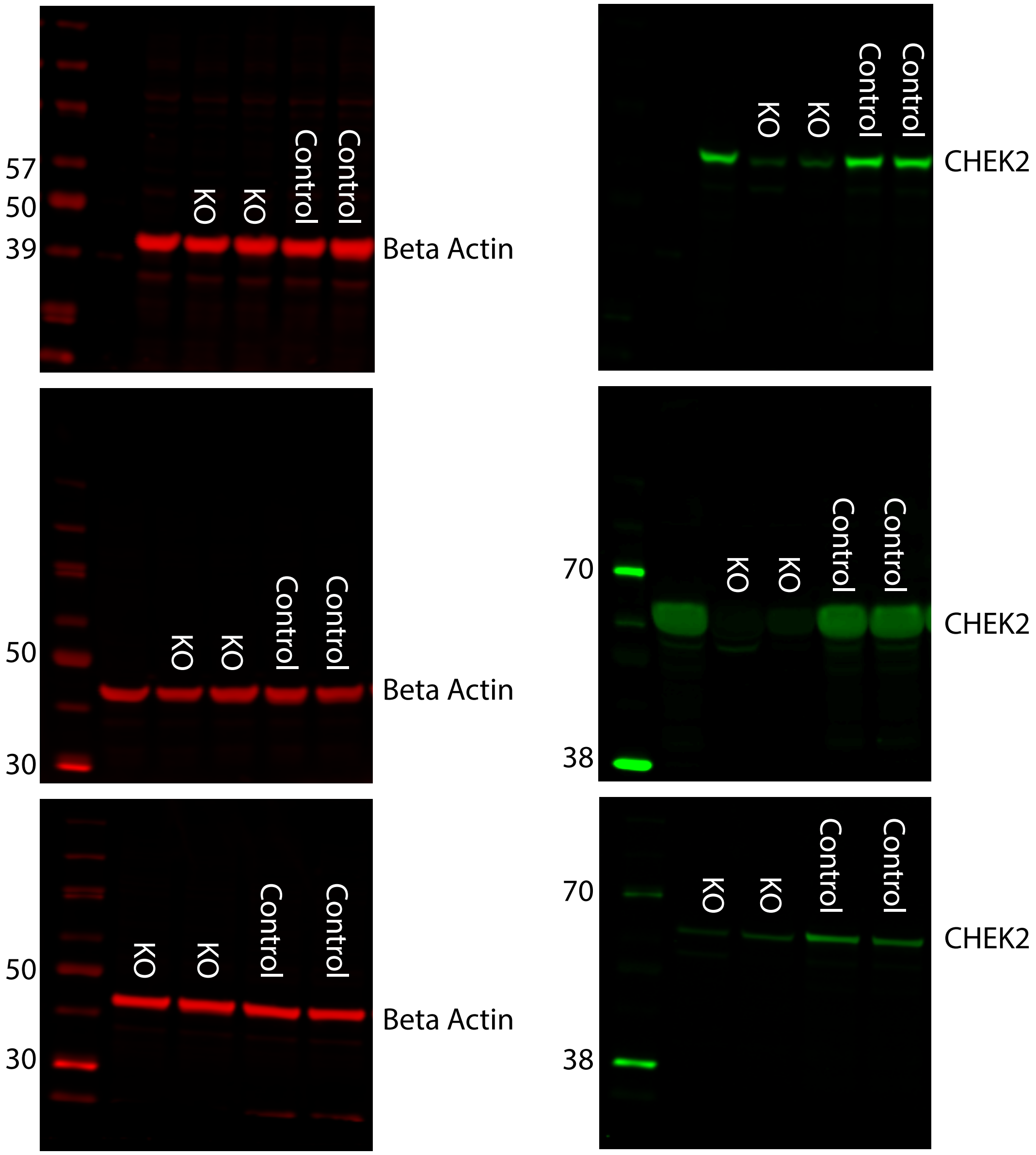


Raw data for Western blots in Figure 4. Uncropped images of western blots displayed in Fig 4a.
